# Entropy analysis on EMHD 3D micropolar tri-hybrid nanofluid flow of solar radiative slendering sheet by a machine learning algorithm

**DOI:** 10.1038/s41598-023-45469-6

**Published:** 2023-11-06

**Authors:** Shaik Jakeer, H. Thameem Basha, Seethi Reddy Reddisekhar Reddy, Mohamed Abbas, Mohammed S. Alqahtani, K. Loganathan, A. Vivek Anand

**Affiliations:** 1 School of Technology, The Apollo University, Chittoor, A.P 517127 India; 2 Department of Mathematical Sciences, Ulsan National Institute of Science and Technology, Ulsan, South Korea; 3 Department of Mathematics, Koneru Lakshmaiah Education Foundation, Bowrampet, Hyderabad, Telangana 500043 India; 4 Electrical Engineering Department, College of Engineering, King Khalid University, 61421 Abha, Saudi Arabia; 5 Radiological Sciences Department, College of Applied Medical Sciences, King Khalid University, 61421 Abha, Saudi Arabia; 6 BioImaging Unit, Space Research Centre, Michael Atiyah Building, University of Leicester, Leicester, LE1 7RH UK; 7 Department of Mathematics and Statistics, Manipal University Jaipur, Jaipur, Rajasthan 303007 India; 8 Department of Aeronautical Engineering, MLR Institute of Technology, Hyderabad, Telangana India

**Keywords:** Engineering, Mathematics and computing

## Abstract

The purpose of this paper is to analyze the heat transfer behavior of the electromagnetic 3D micropolar tri-hybrid nanofluid flow of a solar radiative slendering sheet with non-Fourier heat flux model. The conversion of solar radiation into thermal energy is an area of significant interest as the demand for renewable heat and power continues to grow. Due to their enhanced ability to promote heat transmission, nanofluids can significantly contribute to enhancing the efficiency of solar-thermal systems. The combination of silicon oil-based silicon (Si), magnesium oxide (MgO), and titanium (Ti) nanofluids has attracted attention for their ability to improve the performance of solar-thermal systems. The present study discloses a new approach for intelligent numerical computing solving, which utilizes an MLP feed-forward back-propagation ANN and the Levenberg-Marquard algorithm. The collection of data was conducted for the purpose of testing, certifying, and training the ANN model. The Bvp4c solver in MATLAB is utilized to solve the nonlinear equations governing the momentum, temperature, skin-friction coefficient, and Nusselt number. The characteristics of numerous dimensionless parameters such as porosity parameter $\left(K={0.0,2.0,4.0}\right)$, vortex viscosity parameter $\left({R}_{1}={0.5,1.0,1.5}\right)$, electric field parameter $\left(E={0.0,0.1,0.2}\right)$, thermal relaxation time $\left(\Lambda ={0.01,0.10,0.20}\right)$, heat source/sink parameter, $\left(Q=-{0.3,0.0,0.3}\right)$ thermal radiation parameter $\left(R={0.5,1}.{0,1.5}\right)$, temperature ratio parameter $\left({\theta }_{w}={0.5,1.0,1.5}\right)$,nanoparticle volume fraction $\left(\phi ={0.00,0.02,0.04}\right)$ on Si + MgO + Ti/silicon oil micropolar tri-hybrid nanofluida are analyzed. The ANN model engages in a process of data selection, network construction, training, and evaluation of its effectiveness through the utilization of mean square error. Tables and graphs are used to show how essential parameters affect fluid transport properties. The velocity profile is decreased by higher values of the porosity parameter, whereas the temperature profile is increased. The temperature profile is inversely proportional to higher values of the electric field parameter. The micro-rotation profiles reduced by expanding values vortex viscosity parameter. It has been determined that entropy generation and Bejan number intensifications for enlarged nanoparticle volume fraction.

## Introduction

Natural resources that are regenerated throughout time and are not depleted by human usage are sources of renewable energy. Renewable energy sources have the potential to provide clean, sustainable energy for a number of generations, in contrast to non-renewable energy sources, which depend on limited fossil fuel supplies that will inevitably deplete. The sun's energy may be harnessed using photovoltaic (PV) panels or concentrated solar power (CSP) systems, among other sources of renewable energy^1–4^. Wind energy is the ability to harness the power of the wind to produce electricity. Hydro energy is energy that can be extracted from moving water and used to power hydroelectric power facilities. Geothermal power plants may harness the energy that exists in the heat that exists under the Earth's surface. Biomass energy, which may be used to produce heat and power, is energy derived from organic materials like wood chips, agricultural waste, and landfill gas^5, 6^. Tidal power turbines can harness the energy that comes from the tides' movement. Utilizing wave power equipment, wave energy—the energy from ocean waves—can be harnessed. Each of these renewable energy sources has certain benefits and drawbacks, and how they are used varies according to regional circumstances and the resources that are accessible. This research focuses on solar energy in this aspect. Solar power is a type of renewable energy that is generated by capturing the energy emitted by the sun. The capture and utilization of this form of energy can be achieved through the implementation of photovoltaic (PV) panels or concentrated solar power (CSP) systems. Photovoltaic panels comprise numerous cells that transform sunlight into direct current (DC) electricity. Subsequently, the electricity can be transformed into alternating current (AC) electricity through an inverter, which can be utilized to supply power to residential, commercial, and other electrical devices. Concentrated solar power (CSP) systems employ reflective surfaces such as mirrors or lenses to concentrate the solar radiation onto a limited surface area. This concentrated energy is then utilized to heat a fluid, which in turn powers a turbine to generate electricity. These systems are frequently employed in power plants of significant scale. Solar energy exhibits a diverse array of applications, encompassing various fields. The production of electricity can be achieved through the utilization of solar panels, which can be situated either on the rooftops of buildings or in expansive solar farms. This method is capable of generating electricity for residential, commercial, and communal purposes. Solar thermal systems have the potential to provide water heating for various applications such as residential homes, swimming pools, and other relevant uses. Solar heating and cooling systems have the capability to utilize solar energy for the purpose of regulating the temperature of buildings, either by heating or cooling. The utilization of solar energy in the transportation sector has gained momentum, as evidenced by the increasing popularity of solar-powered vehicles and boats. Additionally, solar panels have been employed to power electric vehicle charging stations. Solar energy has the potential to be utilized in various agricultural applications such as powering irrigation systems and greenhouse heaters. The utilization of solar panels is prevalent in space exploration as a means of providing power to spacecraft, satellites, and rovers. Solar power is an environmentally friendly and sustainable alternative to traditional energy sources that has the capacity to reduce our reliance on non-renewable energy sources and alleviate the emission of greenhouse gases. The anticipated progression of technology is projected to facilitate the proliferation of solar energy applications and utilization^7–10^. Sharma et al.^11^ explored the effects of entropy creation, exchange of heat, and movement of mass using Cu nanoparticles and gyrotactic bacteria as the base fluid, using polyvinyl alcohol-water as the base fluid. Sajid et al.^12^ investigated the behavior of a Maxwell-Sutterby fluid passing over an inclined stretched sheet with changing thermal conductivity, an exponential heat source/sink, and activation energy.

Thermal radiation is a form of electromagnetic radiation that is discharged by any entity that possesses a temperature greater than absolute zero. Thermal energy is exchanged between two objects without any physical interaction or displacement of matter. Solar thermal sheets utilize thermal radiation to harness the sun's energy and transform it into practical heat. The sheets are composed of a material that exhibits high solar radiation absorption characteristics, such as dark-colored metal or ceramic, and are engineered to capture and retain the thermal energy generated^13–16^. Upon exposure to sunlight, the solar thermal sheet undergoes absorption of radiation, resulting in an increase in temperature. Upon being subjected to thermal energy, the material releases thermal radiation that is aimed towards a fluid that is circulated through the sheet, such as air or water^17, 18^. The fluid functions as a heat absorber and facilitates the transfer of thermal energy for the purpose of heating or generating electricity. The efficacy of solar thermal panels is contingent upon multiple variables, such as the composition of the material, the configuration of the panel, and the quantity and quality of solar radiation received. Solar thermal sheets are frequently engineered with specific coatings and reflective surfaces to enhance their efficiency in capturing and retaining solar radiation. Solar thermal sheets are a viable and eco-friendly method for capturing the sun's thermal radiation and transforming it into practical energy for various purposes such as space heating, water heating, and electricity production^19–22^. Salawu et al.^23^ investigated the inner parabola solar trough collector of an airplane wing for the movement rate, temperature fluctuation, and entropy creation of the magnetic Prandtl-Eyring hybrid nanofluid flow. Abbas et al.^24^ investigated how lowered gravity and solar radiation affected fluid movement and heat transfer in a magnetohydrodynamic model with a enclosed inside a porous material is a stable, solid spherical. Sajid et al.^25^ examined the MHD Maxwell nanofluid passing through a Darcy-Forchheimer porous media with nonlinear thermal radiation and variable thermal conductivity.

A nanofluid is a fluid that is made up of nanoparticles, usually within the size range of 1 to 100 nm that are dispersed within a base fluid, such as water, oil, or ethylene glycol. In 1995, Choi introduced the concept of utilizing nanofluids^26^ to augment heat transfer. The observation was made that the inclusion of nanoparticles in a fluid could lead to a substantial increase in its thermal conductivity as compared to the fluid in its pure form. Subsequent to that, there has been a significant surge in the investigation of nanofluids, encompassing a diverse array of nanoparticles and base fluids, with a focus on their thermal characteristics and prospective utilities^27–30^. Hybrid nanofluids represent a contemporary advancement that amalgamates multiple types of nanoparticles within a solitary fluid to augment its thermal conductivity characteristics to a greater extent. Hybrid nanofluids are formulated by selecting distinct nanoparticle varieties based on their complementary attributes, such as their capacity to enhance thermal conductivity, viscosity, and stability. The amalgamation of nanoparticles within a solitary fluid has the potential to produce synergistic outcomes, thereby enhancing the efficiency of heat transfer and diminishing energy consumption across diverse applications^31–33^. Hybrid nanofluids have been the subject of extensive research for their potential utilization in various usages such as preservation of microelectronic devices, solar collectors, automotive engines, and industrial heat exchangers. The term "tri-hybrid" denotes the coexistence of three discrete categories of nanoparticles in the nanofluid. The selection of nanoparticles is contingent upon the intended application and the desired performance improvements, and may encompass a diverse array of materials, including but not limited to metals, metal oxides, or carbon-based nanoparticles. The capacity of heat transfer enhancement and energy consumption reduction has rendered them a highly appealing alternative for numerous industries. Researchers are currently investigating their potential for implementation in novel and developing applications. The present study investigates the suitability of silicon oil-based silicon (Si), magnesium oxide (MgO), and titanium (Ti) nanofluids for deployment in diverse solar thermal applications. The materials silicon (Si), magnesium oxide (MgO), and titanium (Ti) are utilized in a variety of solar applications due to their distinctive properties and functionalities. Silicon is a crucial element in solar energy technology, serving as the fundamental building block for the majority of solar cells. The semiconducting characteristics of the material facilitate the conversion of solar radiation into electrical energy, rendering it a crucial constituent of photovoltaic arrangements. Furthermore, the utilization of silicon-derived silicon dioxide (SiO2) as an anti-reflective coating on solar panels serves to reduce reflection and enhance light absorption, ultimately resulting in heightened energy conversion efficiency. MgO is also a significant contributor to solar applications. Due to its notable thermal conductivity and high heat capacity, it is deemed a fitting material for the purpose of thermal energy storage in concentrated solar power (CSP) facilities. The utilization of MgO allows for the retention of surplus thermal energy during periods of high solar irradiance, thereby facilitating uninterrupted electricity production in instances of reduced sunlight availability. The application of MgO coatings on solar panels confers both corrosion resistance and insulation properties, thereby enhancing their durability and overall efficiency. Titanium (Ti) exhibits a range of solar applications. The semiconductor material utilized in dye-sensitized solar cells (DSSCs) is titanium dioxide (TiO2) nanoparticles, which are responsible for light absorption and subsequent electric current generation. Reflective coatings that integrate titanium are utilized in solar mirrors or concentrators to effectively concentrate sunlight onto a receiver, thereby augmenting the energy yield. In addition, the photocatalytic characteristics of titanium dioxide render it a valuable material for environmental applications that are driven by solar energy, such as the degradation of pollutants and the splitting of water. Islam et al.^34^ used discrete data to determine the heat transfer rate of CuO-Co_3_O_4_ nanoparticles in various shapes when exposed to solar light. Aytaç et al.^35^ investigated the impacts of employing MgO-CuO nanoparticles on energy and exergetic capabilities of a pipe for heating and evacuation via a solar water collector. Sajid et al.^36^ investigated the impact that tetra hybrid nanoparticles have on binary fluids using a unique Hamilton and Crosser model and a catalytic process, with ethanol biofuel used as the base fluid in the study they conducted. Sajid et al.^37^ examined the influence of an extended new tetra hybrid Tiwari and Das nanofluid model on blood flow arteries while also taking into account the Cross non-Newtonian fluid model. Sajid et al.^38^ investigated at the two-dimensional Reiner-Philippoff fluid using unique tetra hybrid nanoparticles and the impacts of thermal radiation and Cattaneo Christov heat flux.

The fundamental goal of the theoretical study is to investigate how the micropolar tri-hybrid nanofluid flow of electromagnetohydrodynamics behaves in terms of heat transfer when uniform heat sources and sinks as well as non-linear thermal radiation are present. In addition, it is noteworthy that the utilization of micropolar hybrid nanofluid comprising silicon oil-based silicon (Si), magnesium oxide (MgO), and titanium (Ti) nanoparticles in conjunction with a non-Fourier heat flux model for analyzing the heat transfer characteristics over a non-Darcy 3-Dimensional slendering sheet has not been explored in the existing literature, to the best of the authors' knowledge. The utilization of base fluid and nanoparticles in the context of solar energy and nuclear reactors has been extensively studied. In particular, studying the phenomenon of fluid flow over a slendering sheet has yielded valuable results that have found practical applications in the field of solar energy, such as in the design of solar aircraft. The Bvp4c solver in MATLAB is utilized to solve the nonlinear equations governing the momentum, temperature, skin-friction coefficient, and Nusselt number. Tables and graphs are used to show how essential parameters affect fluid transport properties. The present study aims to address the following research questions with numerical and ANN procedures to the hybrid nanofliuds:

- How does solar radiation influence the heat transfer and temperature distribution in a micropolar hybrid nanofluid flowing over a 3-dimensional slendering sheet with a non-linear radiation?
- What are the effects of the presence of silicon oil-based silicon (Si), magnesium oxide (MgO), and titanium (Ti) nanoparticles on the transport properties, including viscosity and thermal conductivity, of the nanofluid?
- How do non-Fourier heat flux models impact the temperature profiles and heat transfer rates in the micropolar nanofluid system?
- How can artificial neural network (ANN) models be developed and utilized to predict complex phenomena, such as skin friction and Nusselt number, within the micropolar nanofluid system?
- How does entropy generation within the nanofluid system vary with different factors, including heat transfer, fluid friction, and Joule heating?
- What are the broader implications of this research for the field of fluid dynamics and nanofluid behavior, and how does it contribute to scientific knowledge in these areas?

## Problem formulation

**Figure 1:**
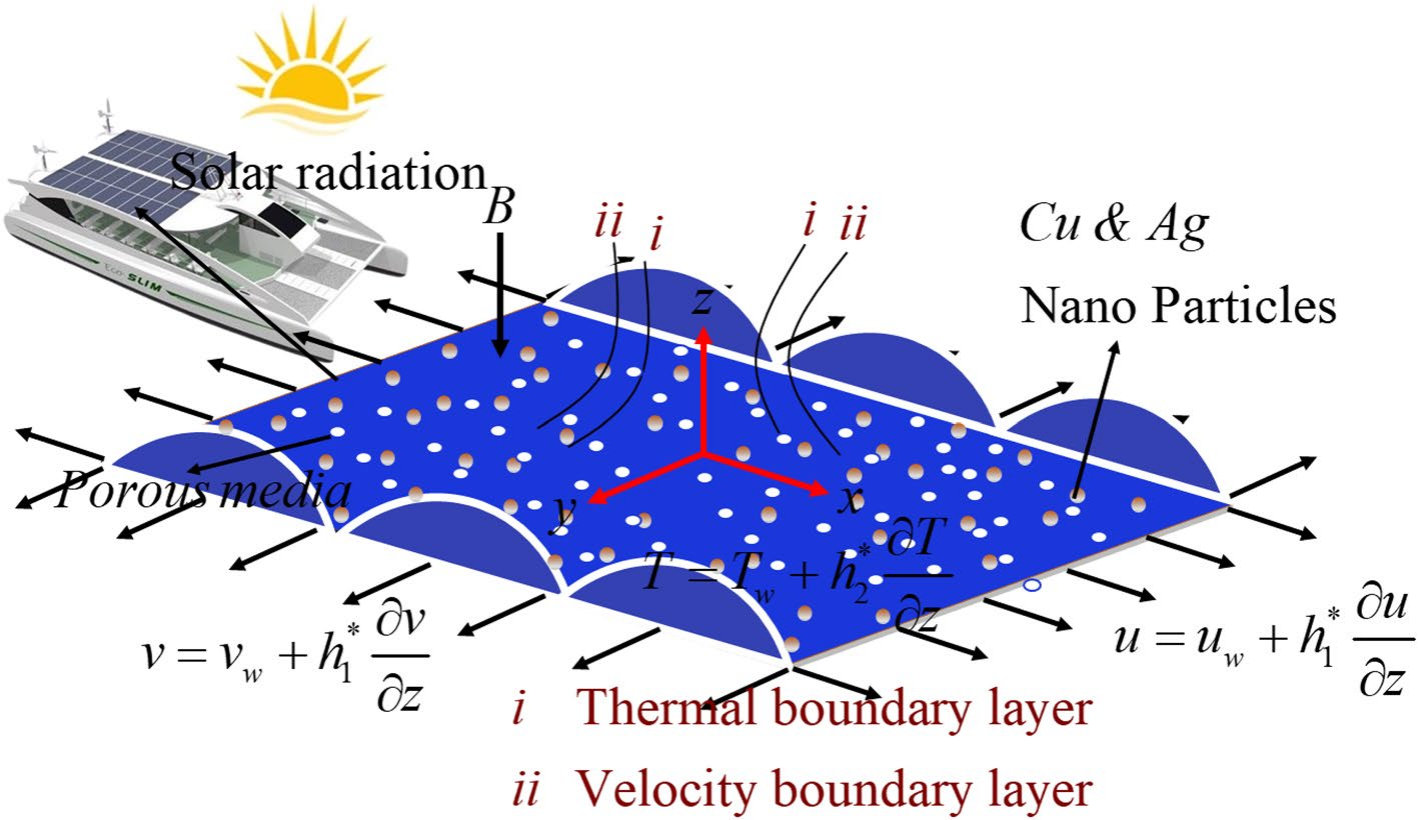
Physical configuration of the problem.

This study aims to examine the properties of a hydromagnetic flow of micropolar hybrid nanofluid that is incompressible and 3-*D*, with magnetic conductivity. A slendering stretchy sheet induces the flow, and multiple slip effects are taken into consideration. The presumption is made that $z=J(c+x+y{)}^{\frac{(1-n)}{2}}$ is a function of n, where n denotes the parameter for the power index of velocity. We made the assumption that *n *≠ 1, indicating that the surface shape results in a flat sheet. The patterns of sheet yields to exterior concave and inner convex are denoted by *n* < 1 and $n>1$, respectively. Furthermore, it is assumed that the value of J is small enough to preserve the sheet's thinness, thereby enabling the pressure gradient along the sheet to be neglected. Given the orientation of the coordinate system, the *x*-axis is directed upwards along the plane, the y-axis is orthogonal to the plane, and the z-axis is orthogonal to the *xy*-plane. The schematic representation of the physical model and its corresponding coordinate system is illustrated in Fig. 1. A perpendicular magnetic field, represented by the symbol *B*, is applied to the plane of the sheet. It is a widely adopted convention to presume that the magnetic Reynolds number is of a considerably low value to disregard the consequences of the induced magnetic field. The boundary layer equations for continuity, momentum, energy, and concentration species can be expressed based on the given assumptions^39–42^.

1 $$\nabla \cdot {\text{V = 0,}}$$

2 $$\rho_{thnf} \left( {{\text{V}} \cdot \nabla {\text{V}}} \right){ = - }\nabla {\text{P + }}\mu_{thnf} \left( {\nabla^{2} {\text{V}}} \right) - \kappa \nabla \times \omega + \left( {J \times B} \right) + R^{*} ,$$

3 $$\rho_{thnf} j\left( {{\text{V}} \cdot \nabla \omega } \right){ = }\chi_{thnf} \left( {\nabla^{2} \omega } \right) - 2\kappa \omega - \kappa \nabla \times {\text{V}},$$

4 $$\left( {\rho c_{p} } \right)_{thnf} \left( {{\text{V}} \cdot \nabla T} \right){ + }\lambda_{T} \left( {\rho c_{p} } \right)_{thnf} \left( {\frac{\partial q}{{\partial t}}{\text{ + V}} \cdot \nabla q - q \cdot \nabla {\text{V + }}\left( {\nabla \cdot {\text{V}}} \right)q} \right){ = }{\text{k}}_{thnf} \left( {\nabla^{2} T} \right) - \nabla \cdot {\text{q}}_{r} + \Phi + Q_{1}^{*} ,$$

In the above equations, ${\text{V}} = \left( {u,v,w} \right)$  is the velocity vector, $\nabla$ is the del or gradient operator, ${\text{P}}$ is the pressure, $\nabla^{2}$ is the is the Laplacian operator, $T$ is the temperature of the fluid, ${\text{k}}_{thnf}$ is the thermal conductivity of the tri-hybrid nanofluid,  $\left( {\rho c_{p} } \right)_{thnf}$ is the effective heat capacity of the tri-hybrid nanofluid, $R^{*}$ is the Forchheimer coefficient, $\Phi$ is the source term representing the dissipation and Joule heating term, $\rho_{thnf}$ is the density of the tri-hybrid nanofluid, $Q_{1}^{*}$ is used to represent internal heat generation density.

The equations presented below represent the standard scaling-based boundary layer approximation in Cartesian form.

5 $$\frac{\partial u}{\partial x}+\frac{\partial v}{\partial y}+\frac{\partial w}{\partial z}=0,$$

6 $$u\frac{\partial u}{\partial x}+v\frac{\partial u}{\partial y}+w\frac{\partial u}{\partial z}=\left(\frac{{\mu }_{thnf}+\kappa }{{\rho }_{thnf}}\right)\frac{{\partial }^{2}u}{\partial {z}^{2}}-\frac{\kappa }{{\rho }_{thnf}}\left(\frac{\partial {\omega }_{2}}{\partial z}\right)-\frac{{\sigma }_{thnf}B}{{\rho }_{thnf}}\left(Bu-{E}_{1}\right) -\frac{{\upsilon }_{thnf}}{{k}_{1}}u-\frac{{F}^{*}}{{k}_{1}}{u}^{2},$$

7 $$u\frac{\partial v}{\partial x}+v\frac{\partial v}{\partial y}+w\frac{\partial v}{\partial z}=\left(\frac{{\mu }_{thnf}+\kappa }{{\rho }_{thnf}}\right)\frac{{\partial }^{2}v}{\partial {z}^{2}}+\frac{\kappa }{{\rho }_{thnf}}\left(\frac{\partial {\omega }_{1}}{\partial z}\right)-\frac{{\sigma }_{thnf}B}{{\rho }_{thnf}}\left(Bv-{E}_{1}\right)-\frac{{\upsilon }_{thnf}}{{k}_{1}}v-\frac{{F}^{*}}{{k}_{1}}{v}^{2},$$

8 $$\left(u\frac{\partial {\omega }_{1}}{\partial x}+v\frac{\partial {\omega }_{1}}{\partial y}+w\frac{\partial {\omega }_{1}}{\partial z}\right){\rho }_{thnf}j={\chi }_{thnf}\frac{{\partial }^{2}{\omega }_{1}}{\partial {z}^{2}}-\kappa \left(2{\omega }_{1}+\frac{\partial v}{\partial z}\right),$$

9 $$\left(u\frac{\partial {\omega }_{2}}{\partial x}+v\frac{\partial {\omega }_{2}}{\partial y}+w\frac{\partial {\omega }_{2}}{\partial z}\right){\rho }_{thnf}j={\chi }_{thnf}\frac{{\partial }^{2}{\omega }_{2}}{\partial {z}^{2}}-\kappa \left(2{\omega }_{2}-\frac{\partial u}{\partial z}\right),$$

10 $$\begin{aligned} & u\frac{\partial T}{\partial x}+v\frac{\partial T}{\partial y}+w\frac{\partial T}{\partial z} \\ & = \frac{{k}_{thnf}}{{\left(\rho {c}_{p}\right)}_{thnf}}\frac{{\partial }^{2}T}{\partial {z}^{2}}-\frac{1}{{\left(\rho {c}_{p}\right)}_{thnf}}\frac{\partial {q}_{r}}{\partial z}+\frac{{Q}^{*}}{{\left(\rho {c}_{p}\right)}_{thnf}}\left(T-{T}_{\infty }\right)+\frac{{\mu }_{thnf}}{{\left(\rho {c}_{p}\right)}_{thnf}}\left({\left(\frac{\partial u}{\partial z}\right)}^{2}+{\left(\frac{\partial v}{\partial z}\right)}^{2}\right)\\ &\qquad+\frac{{\sigma }_{thnf}}{{\left(\rho {c}_{p}\right)}_{thnf}}\left({\left(Bu-{E}_{1}\right)}^{2}+{\left(Bv-{E}_{1}\right)}^{2}\right)+\frac{{\mu }_{hnf}}{{k}_{1}{\left(\rho {c}_{p}\right)}_{thnf}}\left({u}^{2}+{v}^{2}\right)\\ &\qquad-{\lambda }_{T}\left[{u}^{2}\frac{{\partial }^{2}T}{\partial {x}^{2}}+{v}^{2}\frac{{\partial }^{2}T}{\partial {y}^{2}}+{w}^{2}\frac{{\partial }^{2}T}{\partial {z}^{2}}+2uv\frac{{\partial }^{2}T}{\partial x\partial y}+2vw\frac{{\partial }^{2}T}{\partial y\partial z}+2uw\frac{{\partial }^{2}T}{\partial x\partial z}\right.\\ &\qquad\left.+\left(u\frac{\partial u}{\partial x}+v\frac{\partial u}{\partial y}+w\frac{\partial u}{\partial z}\right)\frac{\partial T}{\partial x}+\left(u\frac{\partial v}{\partial x}+v\frac{\partial v}{\partial y}+w\frac{\partial v}{\partial z}\right)\frac{\partial T}{\partial y}\right. \\ &\qquad \left.+\left(u\frac{\partial w}{\partial x}+v\frac{\partial w}{\partial y}+w\frac{\partial w}{\partial z}\right)\frac{\partial T}{\partial z}\right],\end{aligned}$$

The boundary conditions are

$$\begin{aligned} & u={u}_{w}+{h}_{1}^{*}\left(\frac{\partial u}{\partial z}\right),v={v}_{w}+{h}_{1}^{*}\left(\frac{\partial v}{\partial z}\right),w=0,{\omega }_{1}=l\frac{\partial v}{\partial z},{\omega }_{2}=-l\frac{\partial u}{\partial z}, T={T}_{w}+{h}_{2}^{*}\left(\frac{\partial T}{\partial z}\right), \\& \text{at }z=\frac{J}{{\left(x+y+c\right)}^{\frac{n-1}{2}}}, u\to 0,v\to 0,{\omega }_{1}\to 0,{\omega }_{2}\to 0,T\to {T}_{\infty }\text{ as }z\to \infty (7).\end{aligned}$$

where $$h_{2}^{*} = \left( {\frac{2 - b}{b}} \right)\xi_{2} \left( {x + y + c} \right)^{{\left( {1 - n} \right)}{0.5}} ,\,h_{1}^{*} = \left( {\frac{{2 - f_{1} }}{{f_{1} }}} \right)\xi_{1} \left( {x + y + c} \right)^{{\left( {1 - n} \right)}{0.5}} ,\,$$ $${\xi }_{2}=\left(\frac{2{\gamma }_{1}}{{\gamma }_{1}+1}\right)\frac{{\xi }_{1}}{Pr}$$ $$E_{1} = E_{0} \left( {x + y + c} \right)^{{\left( {n - 1} \right)}{0.5}} ,\,$$ $${u}_{w}={U}_{0}{\left(x+y+c\right)}^{n},$$ $$\,v_{w} = U_{1} \left( {x + y + c} \right)^{n} ,$$ $${k}_{1}={k}_{0}{\left(x+y+c\right)}^{-\left(n-1\right)},B={B}_{0}{{\left(x+y+c\right)}^{\left(n-1\right)0.5}},$$ $${T}_{w}={T}_{\infty }+{T}_{0}{{\left(x+y+c\right)}^{\left(1-n\right)0.5}},$$ $${\chi }_{thnf}=\left(\frac{\kappa }{2}+{\mu }_{thnf}\right)j$$, $$j=\frac{2{\upsilon }_{f}}{{u}_{w}}$$ .

The expression mentioned above refers to the individual mechanisms of velocity in the *x*, *y*, and *z* axes, which are denoted by the factors *u*, *v*, and *w*. ${h}_{2}^{*}$ is the jump variable of temperature, ${\mu }_{hnf}$ dynamic viscosity of the tri-hybrid nonofluid, ${\omega }_{1},{\omega }_{2}$ are the components of the microrotation vectors,* T* is the fluid temperature, ${\sigma }_{thnf}$ is the electrical conductivity of the tri-hybrid nanofluid, ${\xi }_{1},{\xi }_{2}$ are the constants (mean free path), ${\rho }_{thnf}$ is the density of the fluid, ${\gamma }_{1}$ is the ratio of specific heats, ${T}_{\infty }$ are the temperature of the ambient fluid, ${h}_{1}^{*}$ is the dimensional velocity slip variable, ${\lambda }_{T}$ thermal relaxation time, $\kappa$ is the vortex viscosity, $l$ boundary parameter, ${\left({c}_{p}\right)}_{thnf}$ is the fluid heat capacity of the tri-hybrid nanofluid, *b* is the thermal accommodation coefficients, ${T}_{0}$ are the reference temperature of fluid, ${k}_{1}$ permeability of the porous medium.

The radiative heat flux is expressed in Eq. (10).

11 $${q}_{r}=-\frac{4{\sigma }^{*}}{3{k}_{1}^{*}}\frac{\partial {T}^{4}}{\partial z},$$

where $k_{1}^{*}$ is the mean absorption coefficient and $\sigma^{*}$ is the Stefan-Boltzmann constant. The temperature variations within the flow are considered excessive for the scope of this study. Consequently, it may not be practical to mitigate the radiative heat flux by extending $T^{4}$ in the Taylor series expansion around *T*_*∞*_ while omitting higher-order terms. Moreover, this truncation has led to a reduction in the accuracy of the thermal radiation's influence. Evaluating the partial rate of change $T^{4}$ with *z* in Eq. (11) using implicit differentiation.

From Eqs. (10) and (11), we get

12 $$\begin{aligned} & u\frac{\partial T}{\partial x}+v\frac{\partial T}{\partial y}+w\frac{\partial T}{\partial z}\\ &=\frac{{k}_{thnf}}{{\left(\rho {c}_{p}\right)}_{thnf}}\frac{{\partial }^{2}T}{\partial {z}^{2}}+\frac{1}{{\left(\rho {c}_{p}\right)}_{thnf}}\frac{\partial }{\partial z}\left(\frac{4{\sigma }^{*}}{3{k}_{1}^{*}}4{T}^{3}\frac{\partial T}{\partial z}\right)+\frac{{Q}^{*}}{{\left(\rho {c}_{p}\right)}_{thnf}}\left(T-{T}_{\infty }\right)\\ &\quad\quad+\frac{{\mu }_{hnf}}{{\left(\rho {c}_{p}\right)}_{thnf}}\left({\left(\frac{\partial u}{\partial z}\right)}^{2}+{\left(\frac{\partial v}{\partial z}\right)}^{2}\right)+\frac{{\sigma }_{thnf}}{{\left(\rho {c}_{p}\right)}_{thnf}}\left({\left(Bu-{E}_{1}\right)}^{2}+{\left(Bv-{E}_{1}\right)}^{2}\right)\\ &\quad\quad+\frac{{\mu }_{hnf}}{{k}_{1}{\left(\rho {c}_{p}\right)}_{thnf}}\left({u}^{2}+{v}^{2}\right)-{\lambda }_{T}\left[{u}^{2}\frac{{\partial }^{2}T}{\partial {x}^{2}}+{v}^{2}\frac{{\partial }^{2}T}{\partial {y}^{2}}+{w}^{2}\frac{{\partial }^{2}T}{\partial {z}^{2}}+2uv\frac{{\partial }^{2}T}{\partial x\partial y}+2vw\frac{{\partial }^{2}T}{\partial y\partial z}+2uw\frac{{\partial }^{2}T}{\partial x\partial z}\right.\\ &\qquad\left.+\left(u\frac{\partial u}{\partial x}+v\frac{\partial u}{\partial y}+w\frac{\partial u}{\partial z}\right)\frac{\partial T}{\partial x}+\left(u\frac{\partial v}{\partial x}+v\frac{\partial v}{\partial y}+w\frac{\partial v}{\partial z}\right)\frac{\partial T}{\partial y}+\left(u\frac{\partial w}{\partial x}+v\frac{\partial w}{\partial y}+w\frac{\partial w}{\partial z}\right)\frac{\partial T}{\partial z}\right],\end{aligned}$$

The aforementioned set of PDE (1)-(8) and their corresponding boundary conditions can be transformed into a system of nonlinear coupled ordinary differential equations through the use of suitable transformations.

13 $$\left.\begin{array}{c}\psi ={\left(\frac{2{\nu }_{f}{U}_{0}}{\left(n+1\right)}\right)}^{0.5}{\left(x+y+c\right)}^{0.5\left(n+1\right)}F\left(\zeta \right),\zeta ={\left(\frac{(n+1){U}_{0}}{2{\nu }_{f}}\right)}^{0.5}{\left(x+y+c\right)}^{0.5\left(n-1\right)}z,\\ u={U}_{0}{\left(x+y+c\right)}^{n}F{\prime}\left(\zeta \right),v={U}_{0}{\left(x+y+c\right)}^{n}G{\prime}\left(\zeta \right),\Theta \left(\zeta \right)=\frac{T-{T}_{\infty }}{{T}_{w}-{T}_{\infty }},\\ w=-{\left(\frac{2{\nu }_{f}{U}_{0}}{\left(n+1\right)}\right)}^{0.5}{\left(x+y+c\right)}^{0.5\left(n-1\right)}\left[\frac{n+1}{2}\left(F\left(\zeta \right)+G\left(\zeta \right)\right)+\frac{n-1}{2}\zeta \left(F{\prime}\left(\zeta \right)+G{\prime}\left(\zeta \right)\right)\right],\\ {\omega }_{2}={\left(\frac{\left(n+1\right){U}_{0}^{3}}{2{\nu }_{f}}\right)}^{0.5}{\left(x+y+c\right)}^{0.5\left(3n-1\right)}{H}_{2}\left(\zeta \right),\\ {\omega }_{1}={\left(\frac{\left(n+1\right){U}_{0}^{3}}{2{\nu }_{f}}\right)}^{0.5}{\left(x+y+c\right)}^{0.5\left(3n-1\right)}{H}_{1}\left(\zeta \right),\end{array}\right\}$$

Here $\psi$ is the stream function and $\zeta$ is the similarity variable. Using Eq. (13) the transformed system of equations are,

14 $$\begin{aligned} & n\left(\frac{d}{d\zeta }F\left(\zeta \right)\right)\left(\frac{d}{d\zeta }F\left(\zeta \right)+\frac{d}{d\zeta }G\left(\zeta \right)\right)-\frac{\left(n+1\right)}{2}\left(\frac{{d}^{2}}{d{\zeta }^{2}}F\left(\zeta \right)\right)\left(F\left(\zeta \right)+G\left(\zeta \right)\right)\\ &\quad = \frac{\left(n+1\right)}{2}\left(\frac{\frac{{\mu }_{thnf}}{{\mu }_{f}}+{R}_{1}}{\frac{{\rho }_{thnf}}{{\rho }_{f}}}\right)\left(\frac{{d}^{3}}{d{\zeta }^{3}}F\left(\zeta \right)\right)-\frac{\frac{{\sigma }_{thnf}}{{\sigma }_{f}}}{\frac{{\rho }_{thnf}}{{\rho }_{f}}}M\left(\frac{d}{d\zeta }F\left(\zeta \right)-E\right)-\frac{\frac{{\mu }_{thnf}}{{\mu }_{f}}}{\frac{{\rho }_{thnf}}{{\rho }_{f}}}K\left(\frac{d}{d\zeta }F\left(\zeta \right)\right)\\ &\qquad-\frac{{F}_{s}}{\frac{{\rho }_{thnf}}{{\rho }_{f}}}{\left(\frac{d}{d\zeta }F\left(\zeta \right)\right)}^{2}-\frac{{R}_{1}}{\frac{{\rho }_{thnf}}{{\rho }_{f}}}\frac{\left(n+1\right)}{2}\left(\frac{d}{d\zeta }{H}_{2}\left(\zeta \right)\right)\end{aligned}$$

15 $$\begin{aligned} & n\left(\frac{d}{d\zeta }G \left(\zeta \right)\right)\left(\frac{d}{d\zeta }F \left(\zeta \right)+\frac{d}{d\zeta }G \left(\zeta \right)\right)-\frac{\left(n+1\right)}{2}\left(\frac{{d}^{2}}{d{\zeta }^{2}}G \left(\zeta \right)\right)\left(F \left(\zeta \right)+G \left(\zeta \right)\right) \\ & \quad = \frac{\left(n+1\right)}{2}\left(\frac{\frac{{\mu }_{thnf}}{{\mu }_{f}}+{R}_{1}}{\frac{{\rho }_{thnf}}{{\rho }_{f}}}\right)\left(\frac{{d}^{3}}{d{\zeta }^{3}}G \left(\zeta \right)\right)-\frac{\frac{{\sigma }_{thnf}}{{\sigma }_{f}}}{\frac{{\rho }_{thnf}}{{\rho }_{f}}}M\left(\frac{d}{d\zeta }G \left(\zeta \right)-E\right)-\frac{\frac{{\mu }_{thnf}}{{\mu }_{f}}}{\frac{{\rho }_{thnf}}{{\rho }_{f}}}K\left(\frac{d}{d\zeta }G \left(\zeta \right)\right) \\ &\quad\quad-\frac{{F}_{s}}{\frac{{\rho }_{thnf}}{{\rho }_{f}}}{\left(\frac{d}{d\zeta }G \left(\zeta \right)\right)}^{2}+\frac{{R}_{1}}{\frac{{\rho }_{thnf}}{{\rho }_{f}}}\frac{\left(n+1\right)}{2}\left(\frac{d}{d\zeta }{H}_{1} \left(\zeta \right)\right)\end{aligned}$$

16 $$\begin{aligned} & \frac{\left(3n-1\right)}{2}{H}_{2}\left(\zeta \right)\left(\frac{d}{d\zeta }F\left(\zeta \right)+\frac{d}{d\zeta }G\left(\zeta \right)\right)-\frac{\left(n+1\right)}{2}\left(\frac{d}{d\zeta }{H}_{2}\left(\zeta \right)\right)\left(F\left(\zeta \right)+G\left(\zeta \right)\right)\\ &\quad=\frac{\left(n+1\right)}{2}{R}_{2}\frac{{\rho }_{f}}{{\rho }_{thnf}}\left(\frac{{d}^{2}}{d{\zeta }^{2}}{H}_{2}\left(\zeta \right)\right)-{R}_{1}{R}_{3}\left(2{H}_{2}\left(\zeta \right)-\frac{{d}^{2}}{d{\zeta }^{2}}F\left(\zeta \right)\right)\end{aligned}$$

17 $$\frac{\left(3n-1\right)}{2}{H}_{2}\left(\zeta \right)\left(\frac{d}{d\zeta }F\left(\zeta \right)+\frac{d}{d\zeta }G\left(\zeta \right)\right)-\frac{\left(n+1\right)}{2}\left(\frac{d}{d\zeta }{H}_{2}\left(\zeta \right)\right)\left(F\left(\zeta \right)+G\left(\zeta \right)\right)=\frac{\left(n+1\right)}{2}{R}_{2}\frac{{\rho }_{f}}{{\rho }_{thnf}}\left(\frac{{d}^{2}}{d{\zeta }^{2}}{H}_{2}\left(\zeta \right)\right)-{R}_{1}{R}_{3}\left(2{H}_{2}\left(\zeta \right)-\frac{{d}^{2}}{d{\zeta }^{2}}F\left(\zeta \right)\right)$$

18 $$\begin{aligned} & -\frac{\left(n+1\right)}{2}\left(\frac{d}{d\zeta }\Theta \left(\zeta \right)\right)\left(F\left(\zeta \right)+G\left(\zeta \right)\right)-\frac{\left(n-1\right)}{2}\Theta \left(\zeta \right)\left(\frac{d}{d\zeta }F\left(\zeta \right)+\frac{d}{d\zeta }G\left(\zeta \right)\right)\\ &\quad=\left(\frac{{k}_{thnf}}{{k}_{f}}+\frac{4}{3}R\right)\frac{{\left(\rho {c}_{p}\right)}_{f}}{{\left(\rho {c}_{p}\right)}_{thnf}}\frac{1}{Pr\frac{\left(n+1\right)}{2}\left(\frac{{d}^{2}}{d{\zeta }^{2}}\Theta \left(\zeta \right)\right)\frac{{\left(\rho {c}_{p}\right)}_{f}}{{\left(\rho {c}_{p}\right)}_{thnf}}\left(\zeta \right)}+\frac{\left(n+1\right)}{2}\frac{{\left(\rho {c}_{p}\right)}_{f}}{{\left(\rho {c}_{p}\right)}_{thnf}}\frac{{\mu }_{thnf}}{{\mu }_{f}}\\ & \qquad Ec\left({\left(\frac{{d}^{2}}{d{\zeta }^{2}}F\left(\zeta \right)\right)}^{2}+{\left(\frac{{d}^{2}}{d{\zeta }^{2}}G\left(\zeta \right)\right)}^{2}\right)+\frac{{\left(\rho {c}_{p}\right)}_{f}}{{\left(\rho {c}_{p}\right)}_{thnf}}\frac{{\sigma }_{thnf}}{{\sigma }_{f}}EcM\left({\left(\frac{d}{d\zeta }F\left(\zeta \right)-E\right)}^{2}+{\left(\frac{d}{d\zeta }G\left(\zeta \right)-E\right)}^{2}\right)\\ & \quad+\frac{{\left(\rho {c}_{p}\right)}_{f}}{{\left(\rho {c}_{p}\right)}_{thnf}}\frac{{\mu }_{thnf}}{{\mu }_{f}}EcK \left({\left(\frac{d}{d\zeta }F\left(\zeta \right)\right)}^{2}+{\left(\frac{d}{d\zeta }G\left(\zeta \right)\right)}^{2}\right)\\ & \quad +\frac{{\left(\rho {c}_{p}\right)}_{f}}{{\left(\rho {c}_{p}\right)}_{thnf}}\frac{1}{Pr\frac{\left(n+1\right)}{2}\frac{4R}{3}\left(\begin{array}{c}3\left({\theta }_{w}-1\right)\left(\Theta \left(\zeta \right)\left(\frac{{d}^{2}}{d{\zeta }^{2}}\Theta \left(\zeta \right)\right)+{\left(\frac{d}{d\zeta }\Theta \left(\zeta \right)\right)}^{2}\right)\\ +3{\left({\theta }_{w}-1\right)}^{2}\left(2\Theta \left(\zeta \right){\left(\frac{d}{d\zeta }\Theta \left(\zeta \right)\right)}^{2}+{\Theta }^{2}\left(\zeta \right)\left(\frac{{d}^{2}}{d{\zeta }^{2}}\Theta \left(\zeta \right)\right)\right)\\ +{\left({\theta }_{w}-1\right)}^{3}\left({\Theta }^{3}\left(\zeta \right)\left(\frac{{d}^{2}}{d{\zeta }^{2}}\Theta \left(\zeta \right)\right)+3{\Theta }^{2}\left(\zeta \right){\left(\frac{d}{d\zeta }\Theta \left(\zeta \right)\right)}^{2}\right)\end{array}\right)}\\ & \quad -\Lambda \left(\begin{array}{c}\frac{{\left(n+1\right)}^{2}}{4}\left(\frac{{d}^{2}}{d{\zeta }^{2}}\Theta \left(\zeta \right)\right){\left(F\left(\zeta \right)+G\left(\zeta \right)\right)}^{2}\\ +\frac{\left(n-1\right)\left(n+1\right)}{4}\left(\frac{{d}^{2}}{d{\zeta }^{2}}F\left(\zeta \right)\right)\Theta \left(\zeta \right)\left(F\left(\zeta \right)+G\left(\zeta \right)\right)\\ +\frac{\left(n-1\right)\left(n+1\right)}{4}\left(\frac{{d}^{2}}{d{\zeta }^{2}}G\left(\zeta \right)\right)\Theta \left(\zeta \right)\left(F\left(\zeta \right)+G\left(\zeta \right)\right)\\ +\left(\begin{array}{c}\frac{{\left(n+1\right)}^{2}}{4}\left(\frac{d}{d\zeta }\Theta \left(\zeta \right)\right)\left(F\left(\zeta \right)+G\left(\zeta \right)\right)\\ -\frac{{\left(n-1\right)}^{2}}{4}\Theta \left(\zeta \right)\left(\frac{d}{d\zeta }F\left(\zeta \right)+\frac{d}{d\zeta }G\left(\zeta \right)\right)\end{array}\right)\left(\frac{d}{d\zeta }F\left(\zeta \right)+\frac{d}{d\zeta }G\left(\zeta \right)\right)\end{array}\right),\end{aligned}$$

The associated border circumstances are

19 $$\left.\begin{array}{c}\left.\begin{array}{c}F(\zeta )=\lambda \left(\frac{1-n}{1+n}\right)\left(1+{\tau }_{1}\frac{{d}^{2}}{d{\zeta }^{2}}F\left(\zeta \right)\right),\frac{d}{d\zeta }F\left(\zeta \right)=1+{\tau }_{1}\frac{{d}^{2}}{d{\zeta }^{2}}F\left(\zeta \right),\\ {H}_{1}(\zeta )\text{=} \, l\frac{{d}^{2}}{d{\zeta }^{2}}G\left(\zeta \right),{H}_{2}(\zeta ) \, \text{=} \, -l\frac{{d}^{2}}{d{\zeta }^{2}}F\left(\zeta \right),\Theta (\zeta )=1+{\tau }_{2}\Theta {\prime}(\zeta ),\\ G(\zeta )=\lambda \left(\frac{1-n}{1+n}\right)\left(1+{\tau }_{1}\frac{{d}^{2}}{d{\zeta }^{2}}G\left(\zeta \right)\right),\frac{d}{d\zeta }G\left(\zeta \right)=\beta +{\tau }_{1}\frac{{d}^{2}}{d{\zeta }^{2}}G\left(\zeta \right),\end{array}\right\}{\text{a}}{\text{t}}\zeta =\lambda ,\\ \frac{d}{d\zeta }F\left(\zeta \right)\to 0,\frac{d}{d\zeta }G\left(\zeta \right)\to 0,{H}_{1}\left(\zeta \right)\to 0,{H}_{2}\left(\zeta \right)\to 0,\Theta \left(\zeta \right)\to 0,{\text{a}}{\text{s}}\zeta \to \infty .\end{array}\right\}$$

Equations (14) - (19) have the domain $\left.\lambda ,\infty \right)$ and are nonlinear. It ought to be translated into $\left.0,\infty \right)$ to simplify the calculations. Therefore, we create a new function.

$$\begin{aligned} & F\left(\zeta \right)=f\left(\zeta -\lambda \right)=f\left(\eta \right),G\left(\zeta \right)=g\left(\zeta -\lambda \right)=g\left(\eta \right),\Theta \left(\zeta \right)=\theta \left(\zeta -\lambda \right)=\theta \left(\eta \right).\\ &\quad {H}_{1}(\zeta )={h}_{1}(\zeta -\lambda )={h}_{1}(\eta ),{H}_{2}(\zeta )={h}_{2}(\zeta -\lambda )={h}_{2}(\eta ),\end{aligned}$$

Equations (14)–(19) therefore become,

20 $$\begin{aligned} & n\left(\frac{d}{d\eta }f\left(\eta \right)\right)\left(\frac{d}{d\eta }f\left(\eta \right)+\frac{d}{d\eta }g\left(\eta \right)\right)-\frac{\left(n+1\right)}{2}\left(\frac{{d}^{2}}{d{\eta }^{2}}f\left(\eta \right)\right)\left(f\left(\eta \right)+g\left(\eta \right)\right) \\ &\quad=\frac{\left(n+1\right)}{2}\left(\frac{\frac{{\mu }_{thnf}}{{\mu }_{f}}+{R}_{1}}{\frac{{\rho }_{thnf}}{{\rho }_{f}}}\right)\left(\frac{{d}^{3}}{d{\eta }^{3}}f\left(\eta \right)\right)-\frac{\frac{{\sigma }_{thnf}}{{\sigma }_{f}}}{\frac{{\rho }_{thnf}}{{\rho }_{f}}}M\left(\frac{d}{d\eta }f\left(\eta \right)-E\right)\\ &\qquad -\frac{\frac{{\mu }_{thnf}}{{\mu }_{f}}}{\frac{{\rho }_{thnf}}{{\rho }_{f}}}K\left(\frac{d}{d\eta }f\left(\eta \right)\right)-\frac{{F}_{s}}{\frac{{\rho }_{thnf}}{{\rho }_{f}}}{\left(\frac{d}{d\eta }f\left(\eta \right)\right)}^{2}-\frac{{R}_{1}}{\frac{{\rho }_{thnf}}{{\rho }_{f}}}\frac{\left(n+1\right)}{2}\left(\frac{d}{d\eta }{h}_{2}\left(\eta \right)\right)\end{aligned}$$

21 $$\begin{aligned} & n\left(\frac{d}{d\eta }g\left(\eta \right)\right)\left(\frac{d}{d\eta }f\left(\eta \right)+\frac{d}{d\eta }g\left(\eta \right)\right)-\frac{\left(n+1\right)}{2}\left(\frac{{d}^{2}}{d{\eta }^{2}}g\left(\eta \right)\right)\left(f\left(\eta \right)+g\left(\eta \right)\right)\\ &\quad=\frac{\left(n+1\right)}{2}\left(\frac{\frac{{\mu }_{thnf}}{{\mu }_{f}}+{R}_{1}}{\frac{{\rho }_{thnf}}{{\rho }_{f}}}\right)\left(\frac{{d}^{3}}{d{\eta }^{3}}g\left(\eta \right)\right)-\frac{\frac{{\sigma }_{thnf}}{{\sigma }_{f}}}{\frac{{\rho }_{thnf}}{{\rho }_{f}}}M\left(\frac{d}{d\eta }g \left(\eta \right)-E\right)\\ & \quad\quad-\frac{\frac{{\mu }_{thnf}}{{\mu }_{f}}}{\frac{{\rho }_{thnf}}{{\rho }_{f}}}K\left(\frac{d}{d\eta }g \left(\eta \right)\right) -\frac{{F}_{s}}{\frac{{\rho }_{thnf}}{{\rho }_{f}}}{\left(\frac{d}{d\eta }g\left(\eta \right)\right)}^{2}+\frac{{R}_{1}}{\frac{{\rho }_{thnf}}{{\rho }_{f}}}\frac{\left(n+1\right)}{2}\left(\frac{d}{d\eta }{h}_{1}\left(\eta \right)\right)\end{aligned}$$

22 $$\begin{aligned} & \frac{\left(3n-1\right)}{2}{h}_{1}\left(\eta \right)\left(\frac{d}{d\eta }f\left(\eta \right)+\frac{d}{d\eta }g\left(\eta \right)\right)-\frac{\left(n+1\right)}{2}\left(\frac{d}{d\eta }{h}_{1}\left(\eta \right)\right)\left(f\left(\eta \right)+g\left(\eta \right)\right)\\ &\quad=\frac{\left(n+1\right)}{2}{R}_{2}\frac{{\rho }_{f}}{{\rho }_{thnf}}\left(\frac{{d}^{2}}{d{\eta }^{2}}{h}_{1}\left(\eta \right)\right)-{R}_{1}{R}_{3}\left(2{h}_{1}\left(\eta \right)+\frac{{d}^{2}}{d{\eta }^{2}}g\left(\eta \right)\right)\end{aligned}$$

23 $$\begin{aligned} & \frac{\left(3n-1\right)}{2}{h}_{2}\left(\eta \right)\left(\frac{d}{d\eta }f\left(\eta \right)+\frac{d}{d\eta }g\left(\eta \right)\right)-\frac{\left(n+1\right)}{2}\left(\frac{d}{d\eta }{h}_{2}\left(\eta \right)\right)\left(f\left(\eta \right)+g\left(\eta \right)\right)\\ &\quad=\frac{\left(n+1\right)}{2}{R}_{2}\frac{{\rho }_{f}}{{\rho }_{thnf}}\left(\frac{{d}^{2}}{d{\eta }^{2}}{h}_{2}\left(\eta \right)\right)-{R}_{1}{R}_{3}\left(2{h}_{2}\left(\eta \right)-\frac{{d}^{2}}{d{\eta }^{2}}f\left(\eta \right)\right)\end{aligned}$$

24 $$\begin{aligned} & -\frac{\left(n+1\right)}{2}\left(\frac{d}{d\eta }\theta \left(\eta \right)\right)\left(f\left(\eta \right)+g\left(\eta \right)\right)-\frac{\left(n-1\right)}{2}\theta \left(\eta \right)\left(\frac{d}{d\eta }f\left(\eta \right)+\frac{d}{d\eta }g\left(\eta \right)\right) \\ & \quad =\left(\frac{{k}_{thnf}}{{k}_{f}}+\frac{4}{3}R\right)\frac{{\left(\rho {c}_{p}\right)}_{f}}{{\left(\rho {c}_{p}\right)}_{thnf}}\frac{1}{Pr\frac{\left(n+1\right)}{2}\left(\frac{{d}^{2}}{d{\eta }^{2}}\theta \left(\eta \right)\right)\frac{{\left(\rho {c}_{p}\right)}_{f}}{{\left(\rho {c}_{p}\right)}_{thnf}}\left(\eta \right)}+\frac{\left(n+1\right)}{2}\frac{{\left(\rho {c}_{p}\right)}_{f}}{{\left(\rho {c}_{p}\right)}_{thnf}}\frac{{\mu }_{thnf}}{{\mu }_{f}}\\ & \qquad Ec\left({\left(\frac{{d}^{2}}{d{\eta }^{2}}f\left(\eta \right)\right)}^{2}+{\left(\frac{{d}^{2}}{d{\eta }^{2}}g\left(\eta \right)\right)}^{2}\right) + \frac{{\left(\rho {c}_{p}\right)}_{f}}{{\left(\rho {c}_{p}\right)}_{thnf}}\frac{{\sigma }_{thnf}}{{\sigma }_{f}}EcM\left({\left(\frac{d}{d\eta }f\left(\eta \right)-E\right)}^{2}+{\left(\frac{d}{d\eta }g\left(\eta \right)-E\right)}^{2}\right)\\ &\qquad +\frac{{\left(\rho {c}_{p}\right)}_{f}}{{\left(\rho {c}_{p}\right)}_{thnf}}\frac{{\mu }_{thnf}}{{\mu }_{f}}EcK\left({\left(\frac{d}{d\eta }f\left(\eta \right)\right)}^{2}+{\left(\frac{d}{d\eta }g\left(\eta \right)\right)}^{2}\right) +\frac{{\left(\rho {c}_{p}\right)}_{f}}{{\left(\rho {c}_{p}\right)}_{thnf}}\\ & \qquad \frac{1}{Pr\frac{\left(n+1\right)}{2}\frac{4R}{3} \left(\begin{array}{c}3\left({\theta }_{w}-1\right)\left(\theta \left(\eta \right)\left(\frac{{d}^{2}}{d{\eta }^{2}}\theta \left(\eta \right)\right)+{\left(\frac{d}{d\eta }\theta \left(\eta \right)\right)}^{2}\right)\\ +3{\left({\theta }_{w}-1\right)}^{2}\left(2\theta \left(\eta \right){\left(\frac{d}{d\eta }\theta \left(\eta \right)\right)}^{2}+{\theta }^{2}\left(\eta \right)\left(\frac{{d}^{2}}{d{\eta }^{2}}\theta \left(\eta \right)\right)\right)\\ +{\left({\theta }_{w}-1\right)}^{3}\left({\theta }^{3}\left(\eta \right)\left(\frac{{d}^{2}}{d{\eta }^{2}}\theta \left(\eta \right)\right)+3{\theta }^{2}\left(\eta \right){\left(\frac{d}{d\eta }\theta \left(\eta \right)\right)}^{2}\right)\end{array}\right)}\\ &\quad-\Lambda \left(\begin{array}{c}\frac{{\left(n+1\right)}^{2}}{4}\left(\frac{{d}^{2}}{d{\eta }^{2}}\theta \left(\eta \right)\right){\left(f\left(\eta \right)+g\left(\eta \right)\right)}^{2}\\ +\frac{\left(n-1\right)\left(n+1\right)}{4}\left(\frac{{d}^{2}}{d{\eta }^{2}}f\left(\eta \right)\right)\theta \left(\eta \right)\left(f\left(\eta \right)+g\left(\eta \right)\right)\\ +\frac{\left(n-1\right)\left(n+1\right)}{4}\left(\frac{{d}^{2}}{d{\eta }^{2}}g\left(\eta \right)\right)\theta \left(\eta \right)\left(f\left(\eta \right)+g\left(\eta \right)\right)\\ +\left(\begin{array}{c}\frac{{\left(n+1\right)}^{2}}{4}\left(\frac{d}{d\eta }\theta \left(\eta \right)\right)\left(f\left(\eta \right)+g\left(\eta \right)\right)\\ -\frac{{\left(n-1\right)}^{2}}{4}\theta \left(\eta \right)\left(\frac{d}{d\eta }f\left(\eta \right)+\frac{d}{d\eta }g\left(\eta \right)\right)\end{array}\right)\left(\frac{d}{d\eta }f\left(\eta \right)+\frac{d}{d\eta }g\left(\eta \right)\right)\end{array}\right),\end{aligned}$$

The boundary conditions are

25 $$\left.\begin{array}{c}\left.\begin{array}{c}f(\eta )=\lambda \left(\frac{1-n}{1+n}\right)\left(1+{\tau }_{1}\frac{{d}^{2}}{d{\eta }^{2}}f\left(\eta \right)\right),\frac{d}{d\eta }f\left(\eta \right)=1+{\tau }_{1}\frac{{d}^{2}}{d{\eta }^{2}}f\left(\eta \right),\\ {h}_{1}(\eta )\text{=} \, l\frac{{d}^{2}}{d{\eta }^{2}}g\left(\eta \right),{h}_{2}(\eta ) \, \text{=} \, -l\frac{{d}^{2}}{d{\eta }^{2}}f\left(\eta \right),\theta (\eta )=1+{\tau }_{2}\frac{d}{d\eta }\theta (\eta ),\\ g(\eta )=\lambda \left(\frac{1-n}{1+n}\right)\left(1+{\tau }_{1}\frac{{d}^{2}}{d{\eta }^{2}}g\left(\eta \right)\right),\frac{d}{d\eta }g\left(\eta \right)=\beta +{\tau }_{1}\frac{{d}^{2}}{d{\eta }^{2}}g\left(\eta \right),\end{array}\right\}{\text{a}}{\text{t}}\eta =0,\\ \frac{d}{d\eta }f\left(\eta \right)\to 0,\frac{d}{d\eta }g\left(\eta \right)\to 0,{h}_{1}\left(\eta \right)\to 0,{h}_{2}\left(\eta \right)\to 0,\theta \left(\eta \right)\to 0,{\text{a}}{\text{s}}\eta \to \infty .\end{array}\right\}$$

where, *M* magnetic parameter, ${\theta }_{w}$ temperature ratio parameter, ${\tau }_{2}$ temperature jump parameter, $Ec$ Eckert number, $E$ is the electric field parameter, *Pr* Prandtl number, ${F}_{s}$ is the inertia coefficient, $R_{1}$ vortex viscosity parameter, *R* is the radiation parameter,$K$ is the porosity perameter, $\Lambda$ thermal relaxation time parameter, ${R}_{3}$ micro-inertia density parameter, $\lambda$ wall thickness variable, ${R}_{2}$ spin gradient viscosity parameter, ${\tau }_{1}$ velocity slip variable, $\beta$ stretching ratio parameter.

The aforementioned parameters are delineated in a subsequent manner:

$$M=\frac{\sigma {B}_{0}^{2}}{{\rho }_{f}{U}_{0}},$$ $${\theta }_{w}=\frac{{T}_{w}}{{T}_{\infty }},$$ $${\tau }_{2}=\left(\frac{2-b}{b}\right){\xi }_{2}{\left(\frac{(n+1){U}_{0}}{2{\nu }_{f}}\right)}^{0.5},$$ $$Ec=\frac{{u}_{w}^{2}}{{c}_{{p}_{f}}\left({T}_{w}-{T}_{\infty }\right)},$$ $$E=\frac{{E}_{0}}{{B}_{0}{u}_{w}},$$ $$\mathit{Pr}=\frac{{\mu }_{f}({c}_{p}{)}_{f}}{{k}_{f}},$$ $${F}_{s}=\frac{{F}^{*}{u}_{w}}{{U}_{0}{k}_{0}},$$ $${R}_{1}=\frac{\kappa }{{\mu }_{f}},$$ $$R=\frac{4{\sigma }^{*}{T}_{\infty }^{3}}{{k}_{f}{k}^{*}},$$ $$K=\frac{{\upsilon }_{f}}{{U}_{0}{k}_{0}},$$ $$\Lambda =\frac{{\lambda }_{T}{u}_{w}}{\left(x+y+c\right)},$$ $${R}_{3}=\frac{{\nu }_{f}(x+y+c)}{{u}_{w}j},$$ $$\lambda =J{\left(\frac{\left(n+1\right){U}_{0}}{2{\nu }_{f}}\right)}^{0.5},$$ $${R}_{2}=\frac{{\chi }_{hnf}}{{\mu }_{f}j},$$ $${\tau }_{1}=\left(\frac{2-{f}_{1}}{{f}_{1}}\right){\xi }_{1}{\left(\frac{(n+1){U}_{0}}{2\nu }\right)}^{0.5},$$ $$\beta =\frac{{U}_{1}}{{U}_{0}},$$

The physical characteristics of the hybrid nanofluid are summarized as follows and the values are given in the Table 1:

**Table 1:** Thermo-physical properties of silicone oil, silica (Si), magnesium oxide (MgO) and titanium (Ti).

	$\rho (kg/m^{3} )$	${c}_{p}(J/kgK)$	$k(W/mK)$	$\sigma (\Omega m{)}^{-1}$
Silicone oil	818	1966	0.1	$1.5\times 1{0}^{-4}$
Si	2650	730	1.5	$5\times 1{0}^{-4}$
MgO	3970	879	30	$8\times 1{0}^{-4}$
Ti	4510	540	20.9	$2.5\times 1{0}^{6}$

Thermal conductivity

$$\begin{aligned} & {\sigma }_{\text{nf}}=\frac{\left({\sigma }_{Si}+2{\sigma }_{bf}-2{\phi }_{1}\left({\sigma }_{bf}-{\sigma }_{Si}\right)\right){\sigma }_{bf}}{{\sigma }_{Si}+2{\sigma }_{bf}+{\phi }_{1}\left({\sigma }_{bf}-{\sigma }_{Si}\right)},{\sigma }_{\text{hnf}}=\frac{\left({\sigma }_{MgO}+2{\sigma }_{nf}-2{\phi }_{2}\left({\sigma }_{nf}-{\sigma }_{MgO}\right)\right){\sigma }_{nf}}{{\sigma }_{MgO}+2{\sigma }_{nf}+{\phi }_{2}\left({\sigma }_{nf}-{\sigma }_{MgO}\right)},\\ &\quad{\sigma }_{\text{thnf}}=\frac{\left({\sigma }_{Ti}+2{\sigma }_{hnf}-2{\phi }_{3}\left({\sigma }_{hnf}-{\sigma }_{Ti}\right)\right){\sigma }_{hnf}}{{\sigma }_{Ti}+2{\sigma }_{hnf}+{\phi }_{3}\left({\sigma }_{hnf}-{\sigma }_{Ti}\right)}.\end{aligned}$$

Electrical conductivity

$${\sigma }_{\text{nf}}=\frac{\left({\sigma }_{Si}+2{\sigma }_{bf}-2{\phi }_{1}\left({\sigma }_{bf}-{\sigma }_{Si}\right)\right){\sigma }_{bf}}{{\sigma }_{Si}+2{\sigma }_{bf}+{\phi }_{1}\left({\sigma }_{bf}-{\sigma }_{Si}\right)},{\sigma }_{\text{hnf}}=\frac{\left({\sigma }_{MgO}+2{\sigma }_{nf}-2{\phi }_{2}\left({\sigma }_{nf}-{\sigma }_{MgO}\right)\right){\sigma }_{nf}}{{\sigma }_{MgO}+2{\sigma }_{nf}+{\phi }_{2}\left({\sigma }_{nf}-{\sigma }_{MgO}\right)},{\sigma }_{\text{thnf}}=\frac{\left({\sigma }_{Ti}+2{\sigma }_{hnf}-2{\phi }_{3}\left({\sigma }_{hnf}-{\sigma }_{Ti}\right)\right){\sigma }_{hnf}}{{\sigma }_{Ti}+2{\sigma }_{hnf}+{\phi }_{3}\left({\sigma }_{hnf}-{\sigma }_{Ti}\right)}.$$

Dynamic viscosity

$$\frac{{\mu }_{\text{thnf}}}{{\mu }_{\text{bf}}}=\frac{1}{{\left(1-{\phi }_{Si}\right)}^{2.5}{\left(1-{\phi }_{MgO}\right)}^{2.5}{\left(1-{\phi }_{Ti}\right)}^{2.5}}$$

Density

$$\frac{{\rho }_{\text{thnf }}}{{\rho }_{\text{bf }}=}=\left(1-{\phi }_{Ti}\right)\left(\left(1-{\phi }_{MgO}\right)\left(1-{\phi }_{Si}+{\phi }_{Si}\frac{{\rho }_{Si}}{{\rho }_{bf}}\right)+{\phi }_{MgO}\frac{{\rho }_{MgO}}{{\rho }_{bf}}\right)+{\phi }_{Ti}\frac{{\rho }_{Ti}}{{\rho }_{bf}}$$

Heat capacitance

$$\frac{{\left(\rho {c}_{p}\right)}_{\text{thnf}}}{{\left(\rho {c}_{p}\right)}_{bf}}=\left(1-{\phi }_{Ti}\right)\left(\left(1-{\phi }_{MgO}\right)\left(1-{\phi }_{Si}+{\phi }_{Si}\frac{{\left(\rho {c}_{p}\right)}_{Si}}{{\left(\rho {c}_{p}\right)}_{bf}}\right)+{\phi }_{MgO}\frac{{\left(\rho {c}_{p}\right)}_{MgO}}{{\left(\rho {c}_{p}\right)}_{bf}}\right)+{\phi }_{Ti}\frac{{\left(\rho {c}_{p}\right)}_{Ti}}{{\left(\rho {c}_{p}\right)}_{bf}}$$

where ${\phi }_{Si},$ ${\phi }_{MgO},$ and ${\phi }_{Ti}$ volume fraction of Silica (Si), Magnesium oxide (MgO), and Titanium (Ti) nanoparticles respectively, $bf$ is the base fluid (silicone oil) respectively.

The pertinent engineering parameters are the Nusselt number at a local level, as well as the skin friction coefficients along the *xy* and *yz* axes. These factors are presented in a non-dimensional format.

26 $$\left.\begin{array}{c}{C}_{fxz}R{e}_{x}^{1/2}=2\left[\left(1-l\right){R}_{1}+\frac{{\mu }_{hnf}}{{\mu }_{f}}\right]{\left(\frac{1+n}{2}\right)}^{0.5}{\left.\frac{{d}^{2}}{d{\eta }^{2}}f(\eta )\right|}_{\eta =0},\\ {C}_{fxz}R{e}_{x}^{1/2}=2\left[\left(1+l\right){R}_{1}+\frac{{\mu }_{hnf}}{{\mu }_{f}}\right]{\left.{\left(\frac{1+n}{2}\right)}^{0.5}\frac{{d}^{2}}{d{\eta }^{2}}g(\eta )\right|}_{\eta =0},\\ {\text{N}}{\text{u}}_{x}{\mathit{Re}}_{x}^{-1/2}=-\left(\frac{4}{3}R{\left[1+\left({\theta }_{w}-1\right)\theta \left(\eta \right)\right]}^{3}+\frac{{k}_{hnf}}{{k}_{f}}\right){\left.{\left(\frac{1+n}{2}\right)}^{0.5}\frac{d}{d\eta }\theta (\eta )\right|}_{\eta =0}.\end{array}\right\}$$

where ${\prime}$ denotes the differentiation with respect to $\eta$ (Eqs. (15)-(20)) and ${\mathit{Re}}_{x}=\frac{{u}_{w}(x+y+c)}{{\nu }_{f}}$ is the local Reynolds number.

### Entropy generation

The entropy generation dimensional form is

27 $$\begin{aligned}{S}_{G} & = \frac{{k}_{f}}{{T}_{\infty }^{2}}\left(\frac{{k}_{thnf}}{{k}_{f}}+\frac{16{\sigma }^{*}{T}^{3}}{3{k}^{*}{k}_{f}}\right){\left(\frac{\partial T}{\partial z}\right)}^{2}+\frac{{\mu }_{thnf}}{{T}_{\infty }}\left({\left(\frac{\partial u}{\partial z}\right)}^{2}+{\left(\frac{\partial v}{\partial z}\right)}^{2}\right)\\ &\quad+\frac{{\sigma }_{thnf}}{{T}_{\infty }}\left({\left(Bu-{E}_{1}\right)}^{2}+{\left(Bv-{E}_{1}\right)}^{2}\right)+\frac{{\mu }_{thnf}}{{k}_{1}{T}_{\infty }}\left({u}^{2}+{v}^{2}\right)\end{aligned}$$

The first, second, third and fourth components of above Eq. (27) are referred to as entropy production owing to heat transfer, entropy production due to fluid friction, entropy generation due to Joule heating and entropy generation due to porous.

$$\begin{aligned}{N}_{G}& ={\alpha }_{1}\left(\frac{{k}_{thnf}}{{k}_{f}}+\frac{4}{3}R{\left(1+\left({\theta }_{w}-1\right)\theta \left(\eta \right)\right)}^{3}\right)\frac{\left(n+1\right)}{2}{\left(\frac{d}{d\eta }\theta \left(\eta \right)\right)}^{2}\\ &\quad+\frac{{\mu }_{thnf}}{{\mu }_{f}}Br\frac{\left(n+1\right)}{2}\left({\left(\frac{{d}^{2}}{d{\eta }^{2}}f\left(\eta \right)\right)}^{2}+{\left(\frac{{d}^{2}}{d{\eta }^{2}}g\left(\eta \right)\right)}^{2}\right)\\ &\quad+\frac{{\mu }_{thnf}}{{\mu }_{f}}KBr\left({\left(\frac{d}{d\eta }f\left(\eta \right)\right)}^{2}+{\left(\frac{d}{d\eta }g\left(\eta \right)\right)}^{2}\right)\\ &\quad+\frac{{\sigma }_{thnf}}{{\sigma }_{f}}MBr\left({\left(\frac{d}{d\eta }f\left(\eta \right)-E\right)}^{2}+{\left(\frac{d}{d\eta }g\left(\eta \right)-E\right)}^{2}\right)\end{aligned}$$

${\alpha }_{1}=\frac{\Delta T}{{T}_{\infty }},Br=\frac{{\mu }_{f}{u}_{w}^{2}}{{k}_{f}\Delta T},{N}_{G}=\frac{{S}_{G}{T}_{\infty }{\upsilon }_{f}\left(x+y+c\right)}{{k}_{f}{u}_{w}\Delta T}$ are the dimensionless ratio variable, Brinkman number and the local entropy generation respectively.

The Bejan number is defined as:

$$Be=\frac{\text{Entropygenerationdue toheat transfer}}{\text{Totalentropygeneration}}$$

$$Be=\frac{{\alpha }_{1}\left(\frac{{k}_{hnf}}{{k}_{f}}+\frac{4}{3}R{\left(1+\left({\theta }_{w}-1\right)\theta \left(\eta \right)\right)}^{3}\right)\frac{\left(n+1\right)}{2}{\left(\frac{d}{d\zeta }\theta \left(\eta \right)\right)}^{2}}{\left(\begin{array}{c}{\alpha }_{1}\left(\frac{{k}_{thnf}}{{k}_{f}}+\frac{4}{3}R{\left(1+\left({\theta }_{w}-1\right)\theta \left(\eta \right)\right)}^{3}\right)\frac{\left(n+1\right)}{2}{\left(\frac{d}{d\eta }\theta \left(\eta \right)\right)}^{2}\\ +\frac{{\mu }_{thnf}}{{\mu }_{f}}Br\frac{\left(n+1\right)}{2}\left({\left(\frac{{d}^{2}}{d{\eta }^{2}}f\left(\eta \right)\right)}^{2}+{\left(\frac{{d}^{2}}{d{\eta }^{2}}g\left(\eta \right)\right)}^{2}\right)+\frac{{\mu }_{thnf}}{{\mu }_{f}}KBr\left({\left(\frac{d}{d\eta }f\left(\eta \right)\right)}^{2}+{\left(\frac{d}{d\eta }g\left(\eta \right)\right)}^{2}\right)\\ +\frac{{\sigma }_{thnf}}{{\sigma }_{f}}MBr\left({\left(\frac{d}{d\eta }f\left(\eta \right)-E\right)}^{2}+{\left(\frac{d}{d\eta }g\left(\eta \right)-E\right)}^{2}\right)\end{array}\right)}$$

The pertinent engineering parameters are the Nusselt number at a local level, as well as the skin friction coefficients along the *xy* and *yz* axes. These factors are presented in a non-dimensional format.

### ANN modelling

Artificial Neural Networks (ANNs) represent a contemporary computational approach inspired by the intricate neural networks found in the human brain. These networks aim to replicate the evolutionary processes observed in the brain, exhibiting comparable performance in tasks involving grouping, learning, classification, prediction, and generalization^43^.

The following points highlight the key advantages of utilizing the Artificial Neural Network methodology:

- ANNs demonstrate remarkable effectiveness and efficiency, even when operating with limited hardware resources.
- The complex task of class-distributed mapping is significantly simplified through the use of artificial neural networks.
- The input vector plays a pivotal role in determining outcomes within the training dataset.
- Weights representing outcomes are acquired through iterative training procedures.

The development of training algorithms and the establishment of inter-neuronal connections yield a diverse range of neural network structures. Stratification of neural networks often emerges from intricate interactions among individual neurons. The ANN methodology comprises three distinct layers: input, hidden, and output layers. These layers receive external information, undergo processing, and subsequently transmit output through the artificial neural network. Notably, the input layer directly forwards information to hidden layer neurons in an unprocessed state, facilitated by interconnected weights, connection lines, and neurons. The system maintains a database to support the training of artificial neural networks (ANNs), storing input values and associated weights. The construction of an artificial neural network is guided by data utilization, considering various factors such as the determination of optimal layer counts and hidden neuron configurations.

The multi-layer perceptron architecture, a form of feed-forward neural network (FFNN), has gained significant popularity as an artificial neural network (ANN) model, and it is currently the most widely used. When it comes to training feed-forward neural networks, alternative techniques have shown lower efficiency compared to the backpropagation method. Backpropagation, with its ability to adjust the weights of individual neurons while calculating the network's output error, stands out as a uniform approach for minimizing the output error.

**Figure 2:**
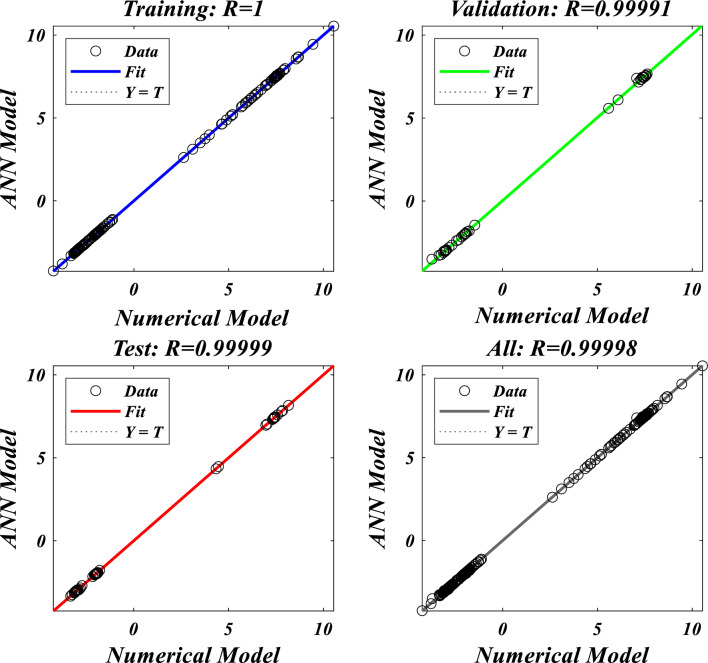
A pictorial illustration of skin friction and the Nusselt number.

The following equation, as shown in Fig. 2, reflects the net input of the jth unknown neuron:

The input layer's *i*th node is symbolized as ${x}_{i}$, the hidden layer's *j*th node is denoted as ${a}_{j}$, and the weight connecting ${x}_{i}$ and ${a}_{j}$ is denoted as $W{1}_{ji}$.

The output's *j*th hidden node is designated as follows:

$${z}_{j}\left(x\right)=\frac{1}{1+{e}^{-{y}_{j}\left(x\right)}},$$

The output layer's *k*th node is designated in the following way:

$${o}_{k}\left(x\right)={\sum }_{j=1}^{m}W{2}_{kj}{z}_{j}+{b}_{k}$$

The weight that connects the output layer's *k*th node to the hidden layer's *j*th node is denoted as $W{2}_{kj}$, where ${b}_{k}$ stands in for the biasing term at the output layer's kth node.

**Figure 3:**
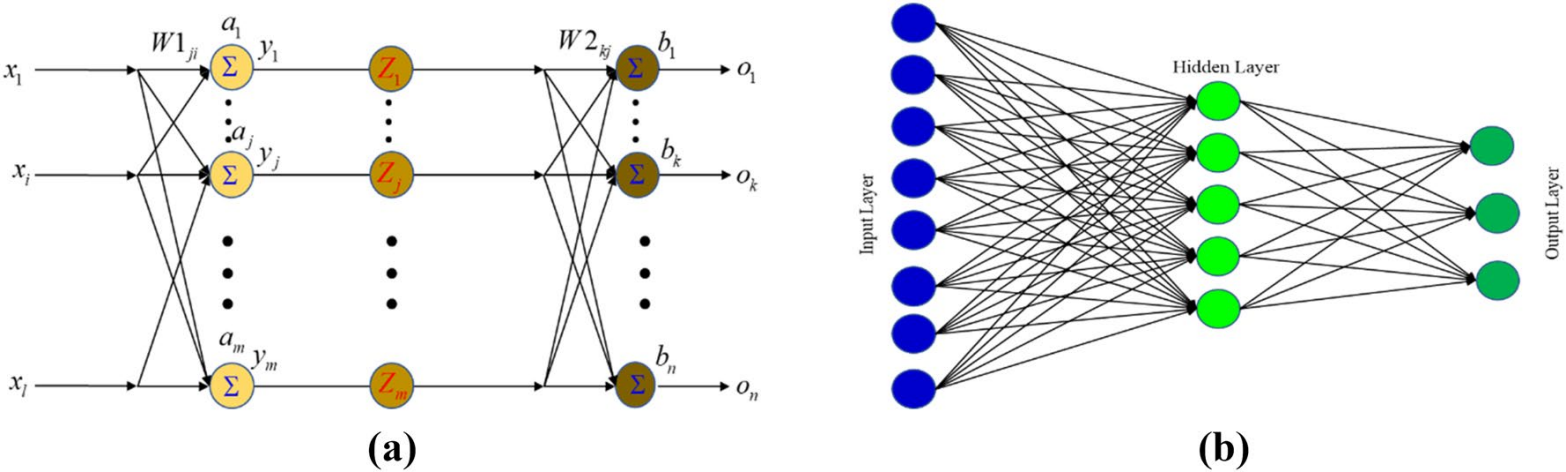
(**a**) Visualization of the neural network's back propagation model, (**b**) Multiple-layer artificial neural network model.

In this study, we focus on quantifying the Nusselt number and skin friction based on the output nodes of an artificial neural network, as depicted in Fig. 3a). We estimate various parameters, including $n,M,\lambda ,l,{R}_{1,}{R}_{2},{R}_{3},E,{F}_{s}$ and $K$, for the input node samples. Determining the optimal number of nodes in the hidden layer of the neural network involves a trial-and-error process, considering factors like the number of training epochs, proper configuration of input parameters, and ensuring a successful learning process. After several iterations, we found that a single hidden layer with five neurons best met our convergence criterion, helping to minimize discrepancies between projected values of ${C}_{fxz}R{e}_{x}^{1/2}$, ${C}_{fxz}R{e}_{x}^{1/2}$ and ${\text{N}}{\text{u}}_{x}{Re}_{x}^{-1/2}$. We divided the dataset into 70% for training, 15% for validation, and another 15% for testing the predictive capabilities of the artificial neural network model, as depicted in Fig. 3b. These components contribute to the ability of the artificial neural network models to capture complex relationships between input and output variables.

The results obtained from the Artificial Neural Network model closely align with the figures obtained through computational analysis. Tables 2 and 3 present the skin friction factor and heat transfer rate porosity parameter $\left(K=\mathrm{0.0,2.0,4.0}\right)$, vortex viscosity parameter $\left({R}_{1}=\mathrm{0.5,1.0,1.5}\right)$, electric field parameter $\left(E=\mathrm{0.0,0.1,0.2}\right)$, thermal relaxation time $\left(\Lambda =\mathrm{0.01,0.10,0.20}\right)$, heat source/sink parameter, $\left(Q=-0.3,\mathrm{0.0,0.3}\right)$ thermal radiation parameter $\left(R=\mathrm{0.5,1.0,1.5}\right)$, temperature ratio parameter $\left({\theta }_{w}=\mathrm{0.5,1.0,1.5}\right)$,nanoparticle volume fraction $\left(\phi =\mathrm{0.00,0.02,0.04}\right)$ values for a range of parameters, including mass concentration of particles, curvature parameter, fluid parameter, magnetic field, melting parameter, thermal radiation parameter, specific heat ratio parameter, and interaction parameter. The tables display these values for various parameter values. The results from the artificial neural network model align well with the quantitative findings. This study demonstrates the ANN's capability to accurately predict both skin friction and Nusselt number.

**Table 2:** Comparative results of ${C}_{fxz}R{e}_{x}^{1/2}$ and ${C}_{fxz}R{e}_{x}^{1/2}$ for various values of $n,M,\lambda ,l,{R}_{1,}{R}_{2},{R}_{3},E,{F}_{s}$ and $K.$

$n$	$M$	$\lambda$	$l$	$R_{1}$	${R}_{2}$	${R}_{3}$	$E$	${F}_{s}$	$K$	${C}_{fxz}R{e}_{x}^{1/2}$			$C_{fyz} Re_{x}^{1/2}$		
										NM	ANN	Error	NM	ANN	Error
0.6	1.0	0.5	0.5	0.5	0.1	0.1	0.01	0.5	0.5	−3.0381	−3.0387	6.24E−04	−2.0121	−2.0156	3.47E−03
0.8	1.0	0.5	0.5	0.5	0.1	0.1	0.01	0.5	0.5	−3.1055	−3.1099	4.38E−03	−2.0587	−2.0640	5.31E−03
1.0	1.0	0.5	0.5	0.5	0.1	0.1	0.01	0.5	0.5	−3.1755	−3.1801	4.58E−03	−2.1071	−2.1116	4.44E−03
1.2	1.0	0.5	0.5	0.5	0.1	0.1	0.01	0.5	0.5	−3.2468	−3.2489	2.13E−03	−2.1566	−2.1582	1.66E−03
0.5	0.0	0.5	0.5	0.5	0.1	0.1	0.01	0.5	0.5	−2.5067	−2.5202	1.34E−02	−1.6450	−1.6527	7.70E−03
0.5	0.2	0.5	0.5	0.5	0.1	0.1	0.01	0.5	0.5	−2.6167	−2.6064	1.02E−02	−1.7206	−1.7130	7.63E−03
0.5	0.4	0.5	0.5	0.5	0.1	0.1	0.01	0.5	0.5	−2.7211	−2.7013	1.98E−02	−1.7928	−1.7796	1.32E−02
0.5	0.6	0.5	0.5	0.5	0.1	0.1	0.01	0.5	0.5	−2.8204	−2.8018	1.86E−02	−1.8616	−1.8503	1.13E−02
0.5	1.0	0.2	0.5	0.5	0.1	0.1	0.01	0.5	0.5	−2.9113	−2.9095	1.74E−03	−1.9242	−1.9248	6.69E−04
0.5	1.0	0.4	0.5	0.5	0.1	0.1	0.01	0.5	0.5	−2.9738	−2.9712	2.59E−03	−1.9676	−1.9689	1.26E−03
0.5	1.0	0.6	0.5	0.5	0.1	0.1	0.01	0.5	0.5	−3.0389	−3.0354	3.45E−03	−2.0127	−2.0134	6.32E−04
0.5	1.0	0.8	0.5	0.5	0.1	0.1	0.01	0.5	0.5	−3.1061	−3.1030	3.04E−03	−2.0593	−2.0591	2.11E−04
0.5	1.0	0.5	0.1	0.5	0.1	0.1	0.01	0.5	0.5	−3.7201	−3.5043	2.16E−01	−1.9252	−1.9441	1.89E−02
0.5	1.0	0.5	0.3	0.5	0.1	0.1	0.01	0.5	0.5	−3.3492	−3.2710	7.82E−02	−1.9589	−1.9681	9.21E−03
0.5	1.0	0.5	0.5	0.5	0.1	0.1	0.01	0.5	0.5	−3.0060	−3.0030	3.08E−03	−1.9900	−1.9910	1.05E−03
0.5	1.0	0.5	0.7	0.5	0.1	0.1	0.01	0.5	0.5	−2.6878	−2.7050	1.73E−02	−2.0188	−2.0165	2.28E−03
0.5	1.0	0.5	0.5	0.2	0.1	0.1	0.01	0.5	0.5	−3.0947	−3.0249	6.97E−02	−1.7512	−1.8024	5.12E−02
0.5	1.0	0.5	0.5	0.4	0.1	0.1	0.01	0.5	0.5	−3.0285	−3.0120	1.65E−02	−1.9151	−1.9264	1.13E−02
0.5	1.0	0.5	0.5	0.6	0.1	0.1	0.01	0.5	0.5	−2.9888	−2.9923	3.44E−03	−2.0609	−2.0573	3.64E−03
0.5	1.0	0.5	0.5	0.8	0.1	0.1	0.01	0.5	0.5	−2.9664	−2.9661	2.32E−04	−2.1928	−2.1942	1.39E−03
0.5	1.0	0.5	0.5	0.5	0.2	0.1	0.01	0.5	0.5	−3.0362	−3.0287	7.50E−03	−2.0096	−2.0086	1.05E−03
0.5	1.0	0.5	0.5	0.5	0.4	0.1	0.01	0.5	0.5	−3.0735	−3.0724	1.14E−03	−2.0342	−2.0375	3.30E−03
0.5	1.0	0.5	0.5	0.5	0.6	0.1	0.01	0.5	0.5	−3.0983	−3.1031	4.77E−03	−2.0506	−2.0559	5.27E−03
0.5	1.0	0.5	0.5	0.5	0.8	0.1	0.01	0.5	0.5	−3.1170	−3.1185	1.44E−03	−2.0630	−2.0617	1.32E−03
0.5	1.0	0.5	0.5	0.5	0.1	0.2	0.01	0.5	0.5	−2.9959	−2.9958	1.17E−04	−1.9832	−1.9852	2.07E−03
0.5	1.0	0.5	0.5	0.5	0.1	0.4	0.01	0.5	0.5	−2.9792	−2.9819	2.64E−03	−1.9720	−1.9732	1.29E−03
0.5	1.0	0.5	0.5	0.5	0.1	0.6	0.01	0.5	0.5	−2.9659	−2.9685	2.60E−03	−1.9630	−1.9616	1.46E−03
0.5	1.0	0.5	0.5	0.5	0.1	0.8	0.01	0.5	0.5	−2.9550	−2.9559	9.26E−04	−1.9557	−1.9524	3.31E−03
0.5	1.0	0.5	0.5	0.5	0.1	0.1	0.10	0.5	0.5	−2.8964	−2.8970	6.10E−04	−1.8211	−1.8146	6.55E−03
0.5	1.0	0.5	0.5	0.5	0.1	0.1	0.20	0.5	0.5	−2.7775	−2.7720	5.56E−03	−1.6393	−1.6243	1.51E−02
0.5	1.0	0.5	0.5	0.5	0.1	0.1	0.30	0.5	0.5	−2.6615	−2.6545	6.99E−03	−1.4628	−1.4531	9.74E−03
0.5	1.0	0.5	0.5	0.5	0.1	0.1	0.40	0.5	0.5	−2.5479	−2.5544	6.54E−03	−1.2907	−1.3040	1.33E−02
0.5	1.0	0.5	0.5	0.5	0.1	0.1	0.01	0.2	0.5	−2.9299	−2.9323	2.40E−03	−1.9646	−1.9635	1.06E−03
0.5	1.0	0.5	0.5	0.5	0.1	0.1	0.01	0.4	0.5	−2.9810	−2.9792	1.78E−03	−1.9816	−1.9823	7.26E−04
0.5	1.0	0.5	0.5	0.5	0.1	0.1	0.01	0.6	0.5	−3.0308	−3.0277	3.07E−03	−1.9983	−1.9992	8.89E−04
0.5	1.0	0.5	0.5	0.5	0.1	0.1	0.01	0.8	0.5	−3.0793	−3.0830	3.71E−03	−2.0150	−2.0136	1.32E−03
0.5	1.0	0.5	0.5	0.5	0.1	0.1	0.01	0.5	0.2	−2.8527	−2.8527	9.81E−06	−1.8800	−1.8830	2.96E−03
0.5	1.0	0.5	0.5	0.5	0.1	0.1	0.01	0.5	0.4	−2.9562	−2.9529	3.30E−03	−1.9543	−1.9552	9.10E−04
0.5	1.0	0.5	0.5	0.5	0.1	0.1	0.01	0.5	0.6	−3.0548	−3.0526	2.15E−03	−2.0247	−2.0263	1.59E−03
0.5	1.0	0.5	0.5	0.5	0.1	0.1	0.01	0.5	0.8	−3.1490	−3.1494	4.13E−04	−2.0917	−2.0945	2.79E−03

**Table 3:** Comparative results of $NuR{e}_{x}^{-1/2}$ for various values of ${R}_{1,}{R}_{2},{R}_{3},E,{F}_{s},K,R,\Lambda ,Ec,{\theta }_{w},Q$ and $\phi .$

$R_{1}$	${R}_{2}$	${R}_{3}$	$E$	${F}_{s}$	$K$	$R$	$\Lambda$	$Ec$	${\theta }_{w}$	$Q$	$\phi$	$NuR{e}_{x}^{-1/2}$		
												NM	ANN	Error
0.2	0.1	0.1	0.01	0.5	0.5	0.5	0.01	0.01	2.0	0.1	0.05	7.2781	7.3137	3.56E−02
0.4	0.1	0.1	0.01	0.5	0.5	0.5	0.01	0.01	2.0	0.1	0.05	7.3988	7.4030	4.16E−03
0.6	0.1	0.1	0.01	0.5	0.5	0.5	0.01	0.01	2.0	0.1	0.05	7.4907	7.4836	7.12E−03
0.8	0.1	0.1	0.01	0.5	0.5	0.5	0.01	0.01	2.0	0.1	0.05	7.5635	7.5658	2.28E−03
0.5	0.2	0.1	0.01	0.5	0.5	0.5	0.01	0.01	2.0	0.1	0.05	7.4511	7.4468	4.33E−03
0.5	0.4	0.1	0.01	0.5	0.5	0.5	0.01	0.01	2.0	0.1	0.05	7.4488	7.4494	6.41E−04
0.5	0.6	0.1	0.01	0.5	0.5	0.5	0.01	0.01	2.0	0.1	0.05	7.4442	7.4471	2.85E−03
0.5	0.8	0.1	0.01	0.5	0.5	0.5	0.01	0.01	2.0	0.1	0.05	7.4397	7.4392	5.12E−04
0.5	0.1	0.2	0.01	0.5	0.5	0.5	0.01	0.01	2.0	0.1	0.05	7.4478	7.4432	4.68E−03
0.5	0.1	0.4	0.01	0.5	0.5	0.5	0.01	0.01	2.0	0.1	0.05	7.4480	7.4435	4.52E−03
0.5	0.1	0.6	0.01	0.5	0.5	0.5	0.01	0.01	2.0	0.1	0.05	7.4480	7.4510	3.02E−03
0.5	0.1	0.8	0.01	0.5	0.5	0.5	0.01	0.01	2.0	0.1	0.05	7.4478	7.4613	1.35E−02
0.5	0.1	0.1	0.1	0.5	0.5	0.5	0.01	0.01	2.0	0.1	0.05	7.5291	7.5676	3.85E−02
0.5	0.1	0.1	0.2	0.5	0.5	0.5	0.01	0.01	2.0	0.1	0.05	7.5910	7.5584	3.26E−02
0.5	0.1	0.1	0.3	0.5	0.5	0.5	0.01	0.01	2.0	0.1	0.05	7.0679	7.4024	3.34E−01
0.5	0.1	0.1	0.4	0.5	0.5	0.5	0.01	0.01	2.0	0.1	0.05	7.3805	7.3894	8.90E−03
0.5	0.1	0.1	0.01	0.2	0.5	0.5	0.01	0.01	2.0	0.1	0.05	7.4700	7.4699	9.10E−05
0.5	0.1	0.1	0.01	0.4	0.5	0.5	0.01	0.01	2.0	0.1	0.05	7.4550	7.4549	1.07E−04
0.5	0.1	0.1	0.01	0.6	0.5	0.5	0.01	0.01	2.0	0.1	0.05	7.4403	7.4338	6.55E−03
0.5	0.1	0.1	0.01	0.8	0.5	0.5	0.01	0.01	2.0	0.1	0.05	7.4259	7.4260	1.39E−05
0.5	0.1	0.1	0.01	0.5	0.2	0.5	0.01	0.01	2.0	0.1	0.05	7.5222	7.5216	6.08E−04
0.5	0.1	0.1	0.01	0.5	0.4	0.5	0.01	0.01	2.0	0.1	0.05	7.4720	7.4687	3.28E−03
0.5	0.1	0.1	0.01	0.5	0.6	0.5	0.01	0.01	2.0	0.1	0.05	7.4238	7.4201	3.74E−03
0.5	0.1	0.1	0.01	0.5	0.8	0.5	0.01	0.01	2.0	0.1	0.05	7.3775	7.3770	5.06E−04
0.5	0.1	0.1	0.01	0.5	0.5	0	0.01	0.01	2.0	0.1	0.05	3.9785	3.9794	8.13E−04
0.5	0.1	0.1	0.01	0.5	0.5	0.2	0.01	0.01	2.0	0.1	0.05	5.6689	5.6661	2.84E−03
0.5	0.1	0.1	0.01	0.5	0.5	0.4	0.01	0.01	2.0	0.1	0.05	6.9346	6.9379	3.37E−03
0.5	0.1	0.1	0.01	0.5	0.5	0.6	0.01	0.01	2.0	0.1	0.05	7.9033	7.8954	7.93E−03
0.5	0.1	0.1	0.01	0.5	0.5	0.5	0.02	0.01	2.0	0.1	0.05	7.4721	7.4723	1.45E−04
0.5	0.1	0.1	0.01	0.5	0.5	0.5	0.03	0.01	2.0	0.1	0.05	7.4967	7.4970	3.09E−04
0.5	0.1	0.1	0.01	0.5	0.5	0.5	0.04	0.01	2.0	0.1	0.05	7.5214	7.5204	9.97E−04
0.5	0.1	0.1	0.01	0.5	0.5	0.5	0.05	0.01	2.0	0.1	0.05	7.5461	7.5445	1.65E−03
0.5	0.1	0.1	0.01	0.5	0.5	0.5	0.01	0.05	2.0	0.1	0.05	6.9655	6.9646	9.04E−04
0.5	0.1	0.1	0.01	0.5	0.5	0.5	0.01	0.1	2.0	0.1	0.05	6.3546	6.3539	7.11E−04
0.5	0.1	0.1	0.01	0.5	0.5	0.5	0.01	0.15	2.0	0.1	0.05	5.7348	5.7346	1.94E−04
0.5	0.1	0.1	0.01	0.5	0.5	0.5	0.01	0.2	2.0	0.1	0.05	5.1067	5.1068	5.93E−05
0.5	0.1	0.1	0.01	0.5	0.5	0.5	0.01	0.01	0.7	0.1	0.05	4.6224	4.6251	2.73E−03
0.5	0.1	0.1	0.01	0.5	0.5	0.5	0.01	0.01	0.9	0.1	0.05	4.8791	4.8771	1.93E−03
0.5	0.1	0.1	0.01	0.5	0.5	0.5	0.01	0.01	1.1	0.1	0.05	5.2008	5.1963	4.49E−03
0.5	0.1	0.1	0.01	0.5	0.5	0.5	0.01	0.01	1.3	0.1	0.05	5.5927	5.5904	2.30E−03
0.5	0.1	0.1	0.01	0.5	0.5	0.5	0.01	0.01	2.0	−0.4	0.05	8.5596	8.5603	6.81E−04
0.5	0.1	0.1	0.01	0.5	0.5	0.5	0.01	0.01	2.0	−0.2	0.05	8.1590	8.1594	3.28E−04
0.5	0.1	0.1	0.01	0.5	0.5	0.5	0.01	0.01	2.0	0.0	0.05	7.7022	7.6994	2.88E−03
0.5	0.1	0.1	0.01	0.5	0.5	0.5	0.01	0.01	2.0	0.2	0.05	7.1716	7.1672	4.46E−03
0.5	0.1	0.1	0.01	0.5	0.5	0.5	0.01	0.01	2.0	0.1	0.00	7.2297	7.2297	5.31E−05
0.5	0.1	0.1	0.01	0.5	0.5	0.5	0.01	0.01	2.0	0.1	0.01	7.2722	7.2721	7.11E−05
0.5	0.1	0.1	0.01	0.5	0.5	0.5	0.01	0.01	2.0	0.1	0.02	7.3152	7.3099	5.26E−03
0.5	0.1	0.1	0.01	0.5	0.5	0.5	0.01	0.01	2.0	0.1	0.03	7.3587	7.3549	3.86E−03

## Results and discussion

**Figure 4:**
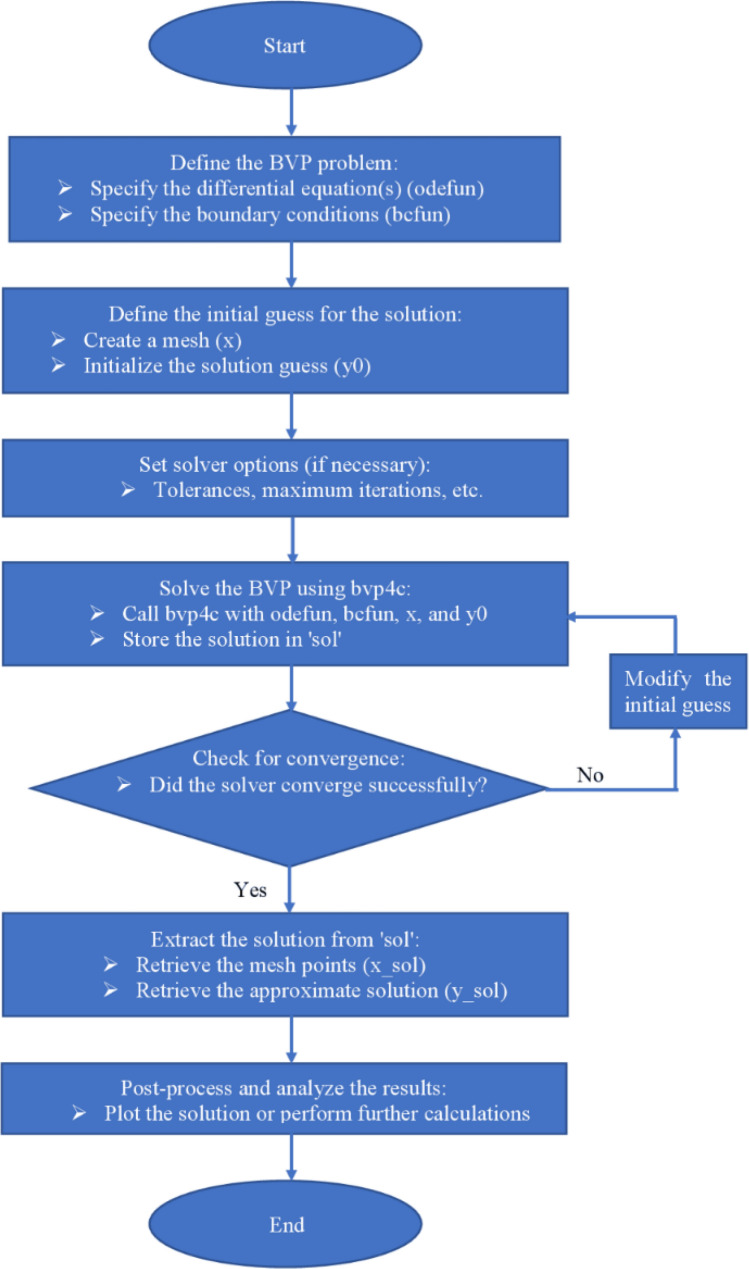
Flow chart of bvp4c scheme.

This section demonstrates entropy analysis using a machine learning technique on an electromagnetic 3D micropolar tri-hybrid nanofluid flow of a solar radiative slendering sheet with non-Fourier heat flux. The present part addresses the importance of momentum and heat considerations in physical phenomena with crucial parameters, including porosity parameter $\left(K=\mathrm{0.0,2.0,4.0}\right)$, vortex viscosity parameter $\left({R}_{1}=\mathrm{0.5,1.0,1.5}\right)$, electric field parameter $\left(E=\mathrm{0.0,0.1,0.2}\right)$, thermal relaxation time $\left(\Lambda =\mathrm{0.01,0.10,0.20}\right)$, heat source/sink parameter, $\left(Q=-\mathrm{0.3,0.0,0.3}\right)$ thermal radiation parameter $\left(R=\mathrm{0.5,1.0,1.5}\right)$, temperature ratio parameter $\left({\theta }_{w}=\mathrm{0.5,1.0,1.5}\right)$,nanoparticle volume fraction $\left(\phi =\mathrm{0.00,0.02,0.04}\right)$ on *Si* + *MgO* + *Ti*/ Silicon oil micropolar tri-hybrid nanofluid velocity $\left({f}{\prime}(\eta )\&{g}{\prime}(\eta )\right)$, micro-rotation $\left({{h}_{1}}{\prime}(\eta )\&{{h}_{2}}{\prime}(\eta )\right)$, temperature $\left(\theta \left(\eta \right)\right)$, entropy generation $\left({N}_{G}\right)$, Bejan number $\left(Be\right)$, skin friction $\left({C}_{fxz}{Re}_{x}^{1/2}\right)$ and Nusselt number $\left(Nu{Re}_{x}^{-1/2}\right)$ for various comparison terms such as convex and inner convex are visualised and elaborately deliberated. The dimensional form of flow and transport equations is resolved by employing the bvp4c solver in MATLAB (Fig. 4), while adhering to particular boundary conditions. Table 4 displays comparative results that demonstrate a significant level of concurrence. This demonstrates that the numerical simulation employed yields precise results.

**Table 4:** Comparison $-f{\prime}{\prime}(0)$ for several values of velocity power index, *n* with $M=0,K=0,E=0,\lambda =0,{\tau }_{1}=0,{F}_{s}=0$ and $\phi =0.$

*n*	10	5	3	2	1	0.5	0	-0.5
Fang et al.^44^	1.0603	1.0486	1.0359	1.0234	1	0.9799	0.9576	1.1667
Khader and Megahed^45^	1.0603	1.0486	1.0358	1.0234	1	0.9798	0.9577	1.1666
Present results	1.06034	1.04862	1.03588	1.02342	1	0.97994	0.95764	1.16666

**Figure 5:**
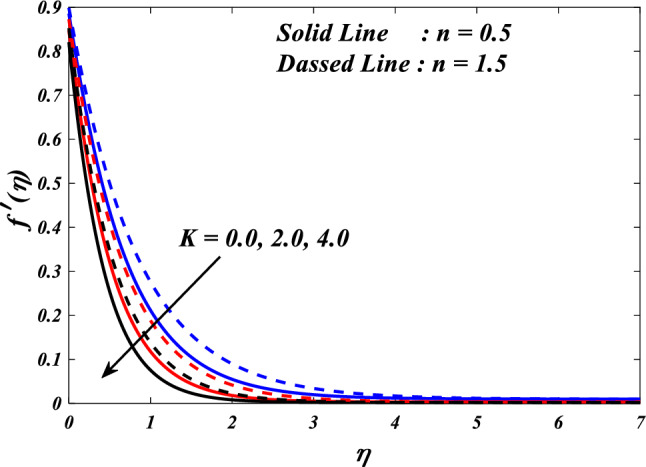
$$f{\prime}(\eta )$$ vs $$K.$$

**Figure 6:**
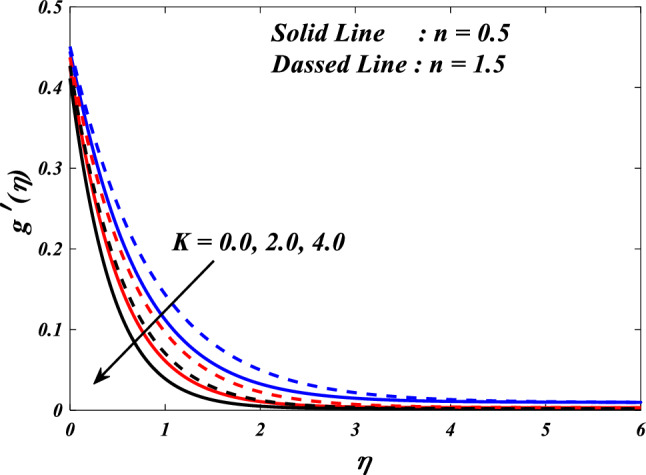
$$g{\prime}(\eta )$$ vs $$K.$$

**Figure 7:**
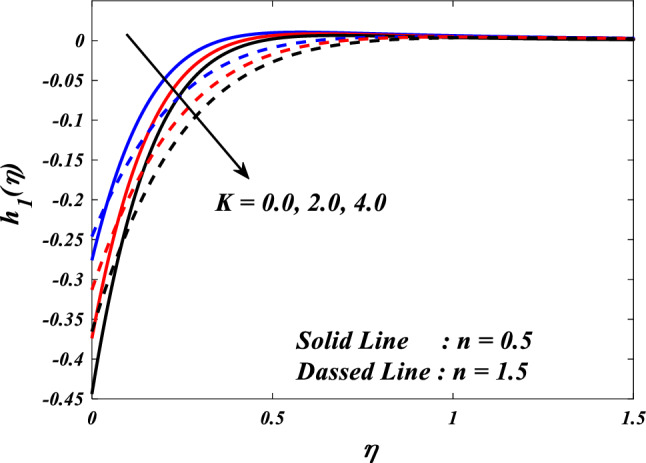
$${h}_{1}(\eta )$$ vs $$K.$$

**Figure 8:**
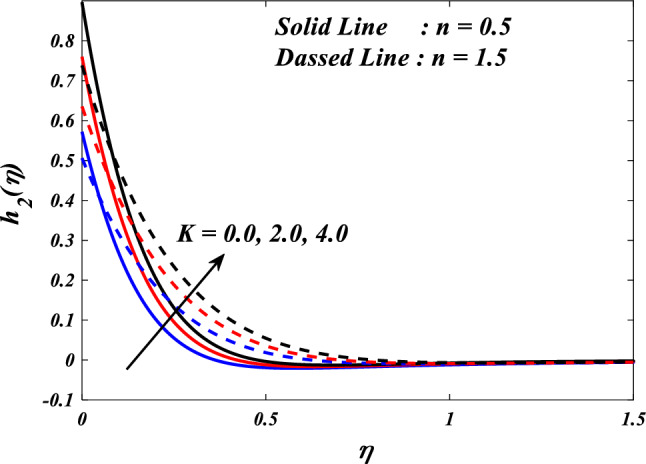
$${h}_{2}(\eta )$$ vs $$K.$$

**Figure 9:**
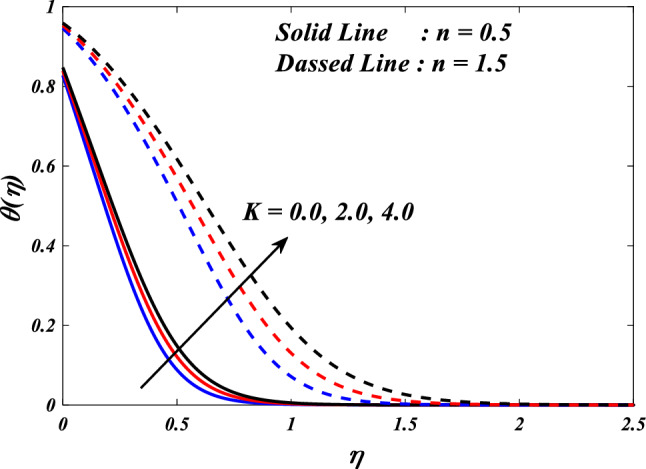
$$\theta (\eta )$$ vs $$K.$$

The effects of the different porosity parameter $\left(K=\mathrm{0.0,2.0,4.0}\right)$ values on velocity $\left({f}{\prime}(\eta )\&{g}{\prime}(\eta )\right)$, micro-rotation $\left({{h}_{1}}{\prime}(\eta )\&{{h}_{2}}{\prime}(\eta )\right)$ and temperature $\left(\theta \left(\eta \right)\right)$ profiles for $n=0.5\&1.5$ are shown in Figs. 5, 6, 7, 8 and 9. Figures 5 and 6 illustrates how the porosity parameter $\left(K=\mathrm{0.0,2.0,4.0}\right)$ influence on velocity profiles $\left({f}{\prime}(\eta )\&{g}{\prime}(\eta )\right)$ changes in $n=0.5\&1.5$, respectively. This graph indicates that increasing $K$ value expansion reduces the velocity profiles $\left({f}{\prime}(\eta )\&{g}{\prime}(\eta )\right)$. Figures 5 and 6 depict the impact of the porosity parameter $\left(K\right)$ on the velocity profiles in $n=0.5\&1.5$. The graph illustrates that an increase in $K$ value expansion leads to a decrease in the velocity profiles of $\left({f}{\prime}(\eta )\&{g}{\prime}(\eta )\right)$. A higher value of the porosity parameter typically indicates a porous medium with a more permeable and interconnected pore structure. Although this phenomenon may facilitate the passage of a greater quantity of fluid, it can also result in heightened flow impedance. The existence of convoluted pathways and diminutive pores may impede the movement of fluids and lead to a reduced velocity profile across the system. Figures 7 and 8 illustrate the observed behavior of $K$ with respect to micro-rotation $\left({{h}_{1}}{\prime}(\eta )\&{{h}_{2}}{\prime}(\eta )\right)$ profiles. It was discovered that ${{h}_{1}}{\prime}(\eta )$ intensifications for enlarged $K$ and diminutions for ${{h}_{2}}{\prime}(\eta )$. A higher value of the porosity parameter typically signifies an increased quantity of empty spaces present within the porous medium. This implies that the solid particles or grains are distributed over a wider area. An increase in porosity can result in a reduction in the frequency or intensity of interactions between the fluid and the solid particles. The diminution of frictional interaction can result in a reduction of micro-rotation of fluid elements due to encountering a lower number of obstacles or interparticle interactions within the porous medium. The relationship between the temperature $\left(\theta \left(\eta \right)\right)$ and the porosity parameter $\left(K\right)$ is shown in Fig. 9. Based on the graph, it is evident that there exists an upward relationship between the temperature $\left(\theta \left(\eta \right)\right)$ and the increasing values of the variable $K$. An increased value of the porosity parameter generally signifies a greater quantity of empty spaces present in the material. The augmentation of the void space can lead to a greater surface area that is accessible for the purpose of heat transfer. Increased surface area in contact with the surrounding medium presents a heightened potential for heat exchange. As a result, it is possible for the temperature profile to experience an increase as a result of heightened heat transfer.

**Figure 10:**
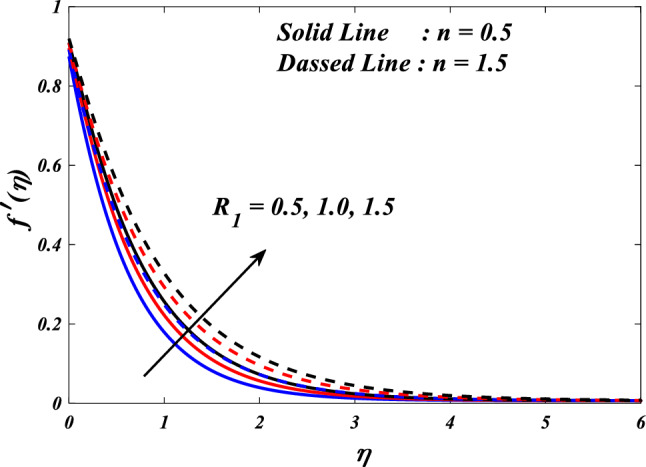
$$f{\prime}(\eta )$$ vs $${R}_{1}.$$

**Figure 11:**
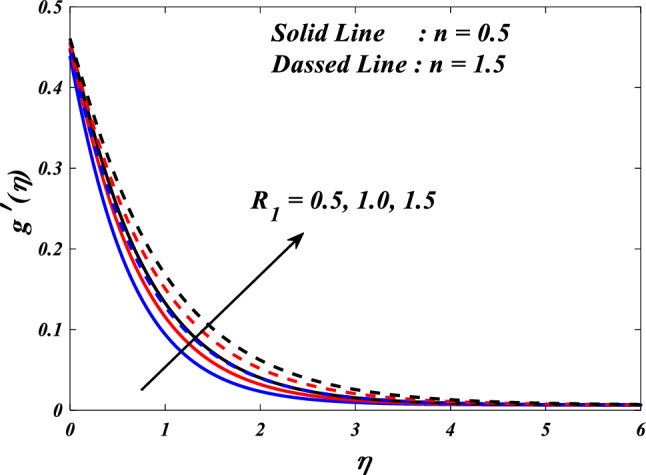
$$g{\prime}(\eta )$$ vs $${R}_{1}.$$

**Figure 12:**
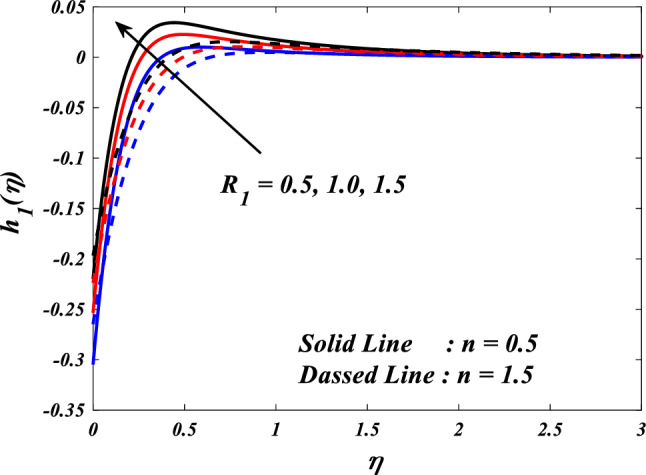
$${h}_{1}(\eta )$$ vs $${R}_{1}.$$

**Figure 13:**
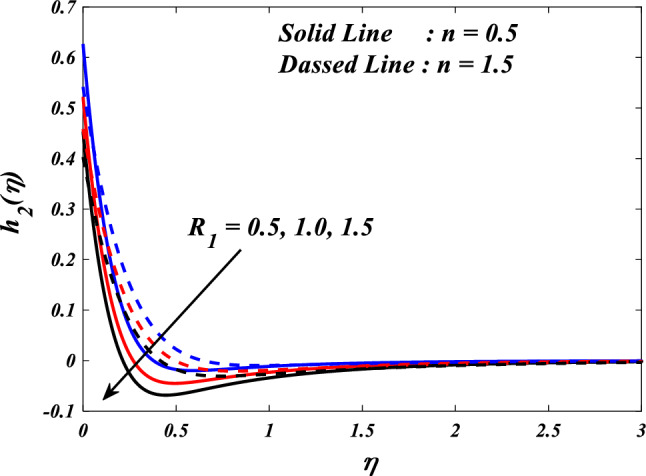
$${h}_{2}(\eta )$$ vs $${R}_{1}.$$

**Figure 14:**
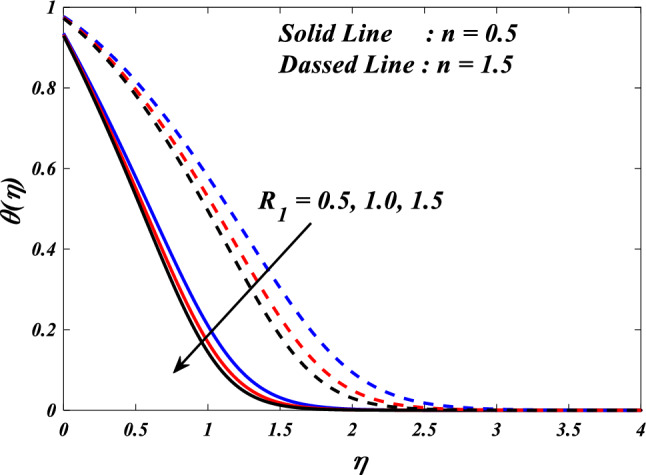
$$\theta (\eta )$$ vs $${R}_{1}.$$

Figures 10, 11, 12, 13 and 14 describe variations in the velocity $\left({f}{\prime}(\eta )\&{g}{\prime}(\eta )\right)$, micro-rotation $\left({{h}_{1}}{\prime}(\eta )\&{{h}_{2}}{\prime}(\eta )\right)$ and temperature $\left(\theta \left(\eta \right)\right)$ profiles influenced by the vortex viscosity parameter $\left({R}_{1}\right)$ for $n=0.5\&1.5$. Figures 10 and 11 demonstrate the influence of the vortex viscosity parameter $\left({R}_{1}\right)$ on the fluctuations in the velocity profile $\left({f}{\prime}(\eta )\&{g}{\prime}(\eta )\right)$ at $n=0.5\&1.5$. The graphical representation illustrates that the velocity profile identified by the parameter $n=0.5\&1.5$ experiences an augmentation as a result of the expansion of values within the ${R}_{1}$. The concept of vortex viscosity pertains to the capacity of fluid constituents to facilitate the dissemination of momentum throughout the fluidic motion. Increased levels of kinematic viscosity lead to an augmented manifestation of momentum diffusion, resulting in a greater uniformity of momentum distribution throughout the fluid. As a result, the gradients of velocity experience an increment, resulting in a greater uniformity in the distribution of velocity. Figures 12 and 13 illustrate the effect of the vortex viscosity parameter $\left({R}_{1}\right)$ on micro-rotation $\left({{h}_{1}}{\prime}(\eta )\&{{h}_{2}}{\prime}(\eta )\right)$ in $n=0.5\&1.5$. The findings indicate that there were increases in ${{h}_{1}}{\prime}(\eta )$ for enlarged ${R}_{1}$ and decrease in ${{h}_{2}}{\prime}(\eta )$. Viscous dissipation refers to the transformation of kinetic energy into thermal energy due to the internal resistance present within the fluid. In the context of micro-rotation, a reduction in the vortex viscosity parameter could indicate a reduction in the dissipation of energy caused by viscosity. As the parameter decreases, the micro-rotation experiences a decrease in dissipation and retains a higher percentage of its initial mechanical energy. The relationship between the temperature $\left(\theta \left(\eta \right)\right)$ and the vortex viscosity parameter $\left({R}_{1}\right)$ is shown in Fig. 14. Based on the graph, it is evident that there exists an downward relationship between the temperature $\left(\theta \left(\eta \right)\right)$ and the increasing values of the variable ${R}_{1}$. An increase in viscosity corresponds to an increase in internal friction, leading to a greater dissipation of energy and subsequent elevation in temperature. Consequently, a rise in the vortex viscosity parameter, assuming it denotes a surge in fluid viscosity, would result in a reduction in the temperature profile.

**Figure 15:**
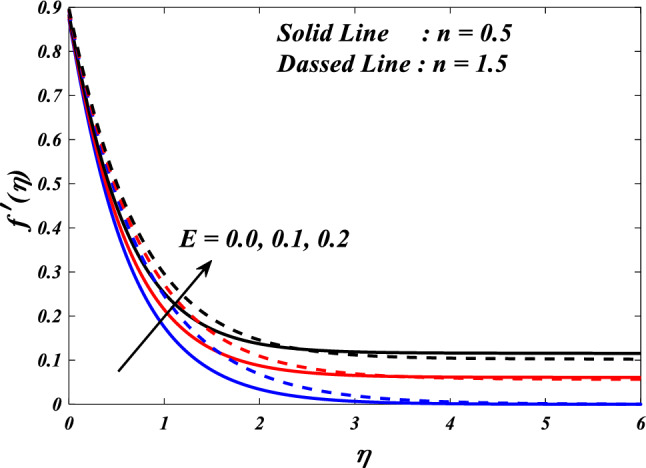
$$f{\prime}(\eta )$$ vs $$E.$$

**Figure 16:**
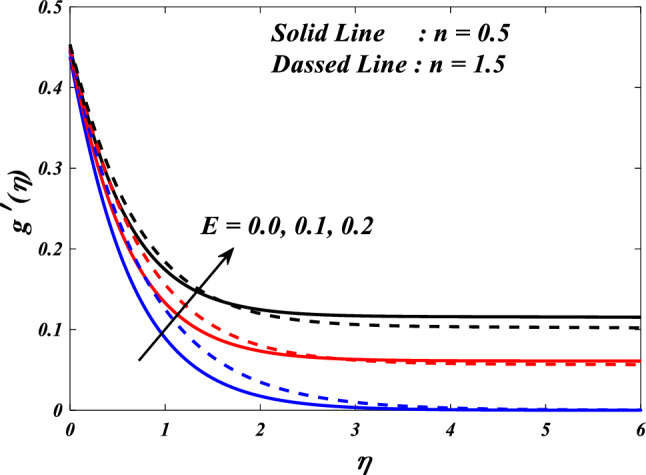
$$g{\prime}(\eta )$$ vs $$E.$$

**Figure 17:**
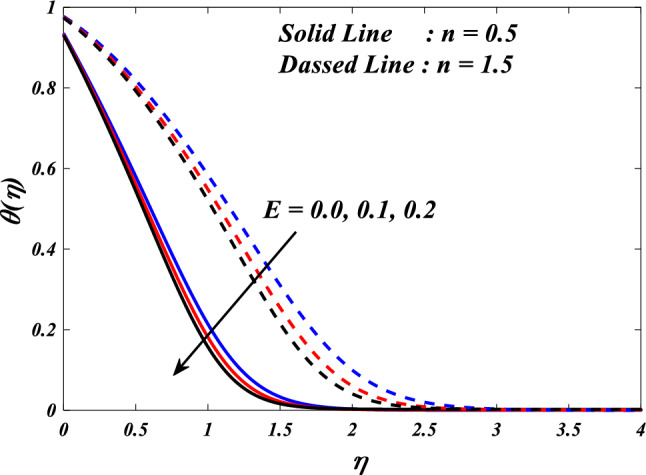
$$\theta (\eta )$$ vs $$E.$$

Figures 15, 16 and 17 illustrate the influence of the electric field parameter $\left(E\right)$, on velocity $\left({f}{\prime}(\eta )\&{g}{\prime}(\eta )\right)$ and temperature $\left(\theta \left(\eta \right)\right)$ profiles changes in $n=0.5\&1.5$, respectively. Figures 15 and 16 demonstrate the influence of the electric field parameter $\left(E\right)$ on the fluctuations in the velocity profile $\left({f}{\prime}(\eta )\&{g}{\prime}(\eta )\right)$ at $n=0.5\&1.5$. This graph indicates that increasing $E$ value expansion increases the velocity profiles $\left({f}{\prime}(\eta )\&{g}{\prime}(\eta )\right)$. The phenomenon of electrokinetics can explain the physical rationale behind the increase in velocity profile with higher values of the electric field parameter. The application of an electric field to a conductive fluid or a fluid containing charged particles has the potential to elicit motion and consequent alterations to the velocity profile. Figure 17 illustrations the consequence of different electric field parameter $\left(E\right)$ on the $\theta \left(\eta \right)$ profile in the case of $n=0.5\&1.5$. It is acknowledged that the $\left(\theta \left(\eta \right)\right)$ decrease when the electric field parameter $E$ increases. It is noteworthy that the resistive heating phenomenon constitutes a fundamental facet of the interactions between electric fields and conductive materials. Typically, it results in a rise in temperature as opposed to a decline. Hence, the observed correlation between the electric field parameter and the temperature profile can be attributed to the resistive heating phenomenon.

**Figure 18:**
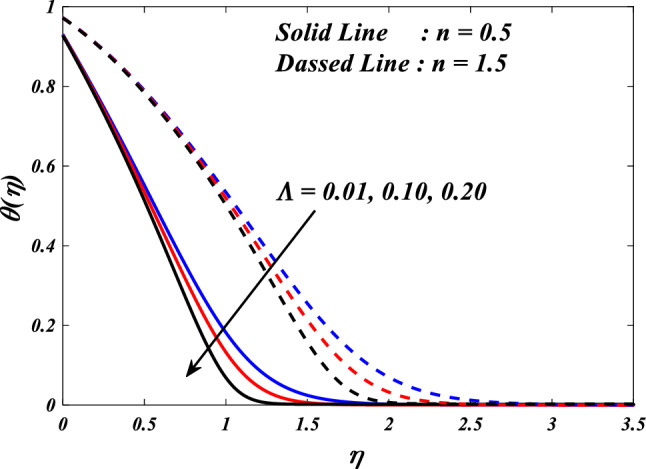
$$\theta (\eta )$$ vs $$\Lambda .$$

**Figure 19:**
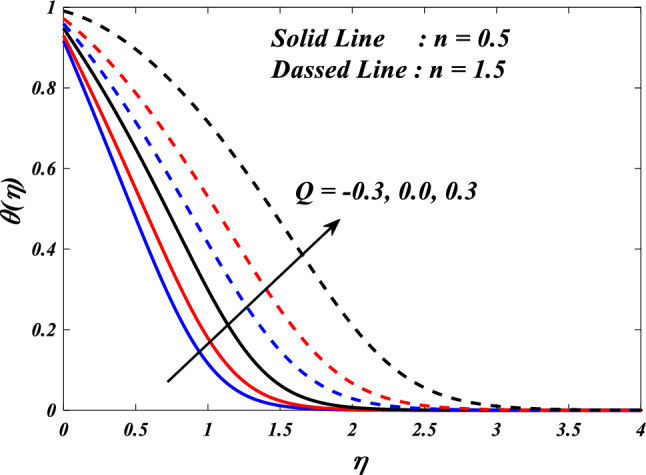
$$\theta (\eta )$$ vs $$Q.$$

**Figure 20:**
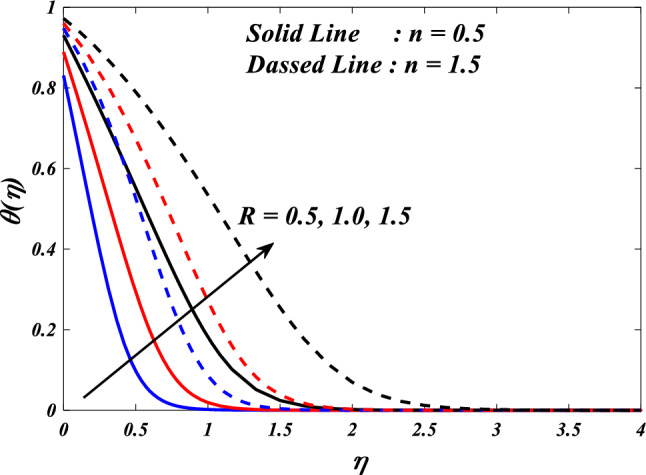
$$\theta (\eta )$$ vs $$R.$$

**Figure 21:**
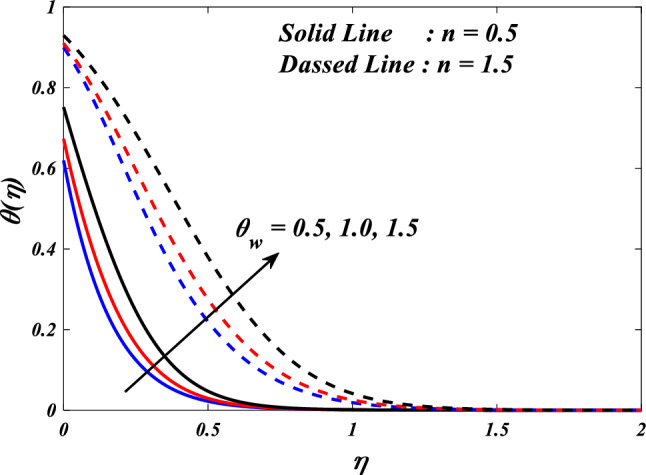
$$\theta (\eta )$$ vs $${\theta }_{w}$$.

Figure 18 depicts the influence of varying thermal relaxation time $\left(\Lambda \right)$ on the temperature profile for $n=0.5\&1.5$. The relationship between temperature $\left(\theta \left(\eta \right)\right)$ and thermal relaxation time $\left(\Lambda \right)$ is recognized to be such that an increase in $\Lambda$ results in a decrease in $\left(\theta \left(\eta \right)\right)$. The thermal relaxation parameter increases proportionally with the duration required for the system to attain thermal equilibrium or respond to variations in temperature. In this scenario, the system may exhibit decelerated temperature variations, leading to a gradual temperature transition throughout the system. Consequently, the temperature profile may exhibit a longer and more gradual shift in temperature from one location to another. Figure 19 illustrates how the heat source/sink parameter $\left(Q\right)$ influence on temperature $\left(\theta \left(\eta \right)\right)$ changes for $n=0.5\&1.5$. According to this graph, the temperature $\left(\theta \left(\eta \right)\right)$ is growths by increasing values in the $Q$. A transfer of thermal energy into a system is facilitated by a heat source. A plausible scenario involves a heat source that is confined to a specific area, such as a heating element that emits thermal energy. As the parameter pertaining to the heat source is augmented, the quantity of thermal energy introduced to the system is amplified, resulting in a rise in temperature. The rise in temperature has the potential to spread throughout the medium, leading to an elevated temperature profile. Figure 20 illustrates the correlation between the thermal radiation parameter $\left(R\right)$ and the temperature $\left(\theta \left(\eta \right)\right)$. According to the graphical representation, it is evident that a direct relationship exists between the temperature $\left(\theta \left(\eta \right)\right)$ and the ascending values of the variable $R$. A direct relationship can be observed between higher temperatures and increased thickness of thermal boundary layers, as well as greater thermal radiation parameters. Figure 21 depicts the impact of the temperature ratio parameter $\left({\theta }_{w}\right)$ on the temperature $\left(\theta \left(\eta \right)\right)$. As per the graph, it can be observed that there is a positive correlation between the temperature $\left(\theta \left(\eta \right)\right)$ and the increasing values of the variable ${\theta }_{w}$. The parameter of temperature ratio signifies the comparative temperatures among distinct regions or constituents of a given system. An increase in the temperature ratio indicates a thermal gradient between two regions or components, with one being comparatively hotter than the other. The temperature differential has an impact on the energy equilibrium within the system. The temperature profile experiences an increase as heat is transferred from a hotter region to a cooler region, ultimately leading to the attainment of thermal equilibrium.

**Figure 22:**
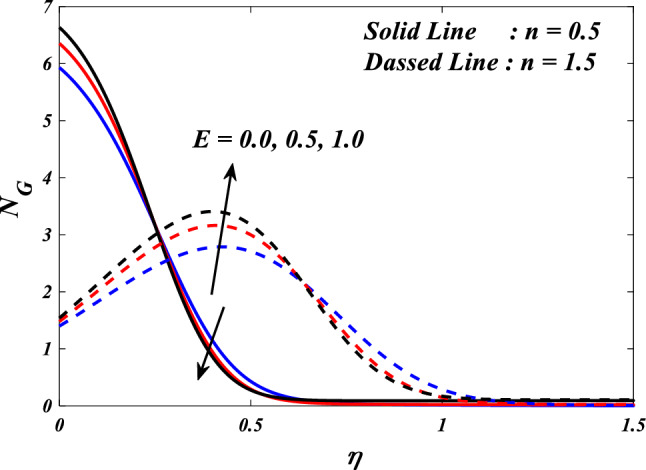
$${N}_{G}$$ vs $$E$$.

**Figure 23:**
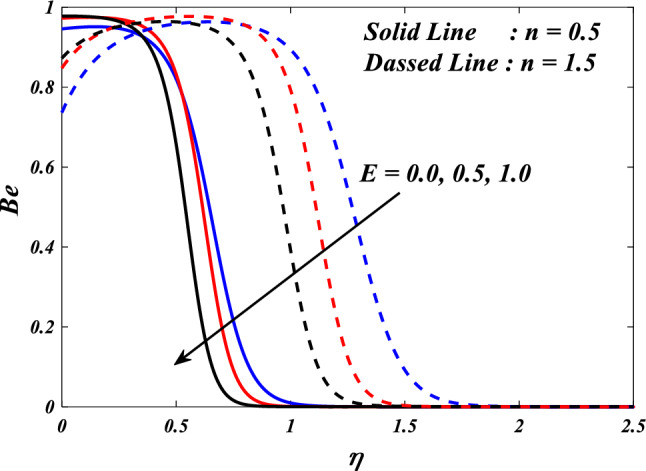
$$Be$$ vs $$E$$.

**Figure 24:**
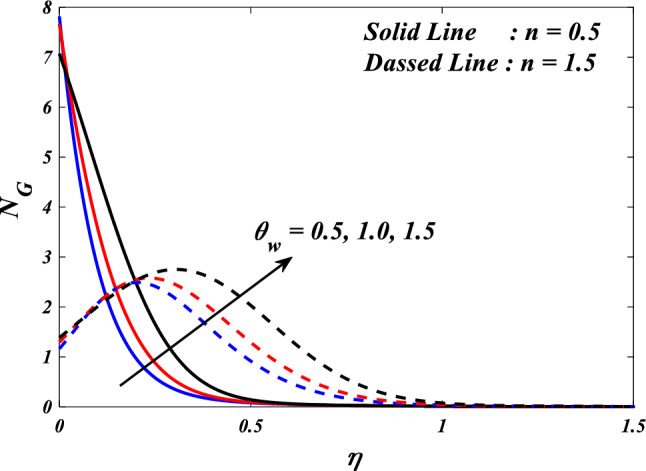
$${N}_{G}$$ vs $${\theta }_{w}$$.

**Figure 25:**
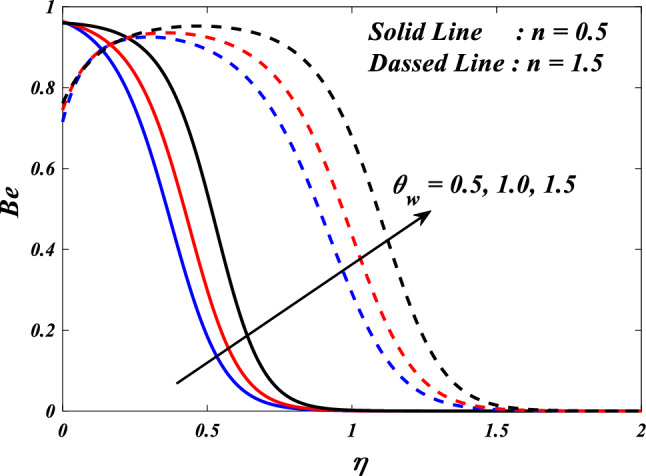
$$Be$$ vs $${\theta }_{w}$$.

**Figure 26:**
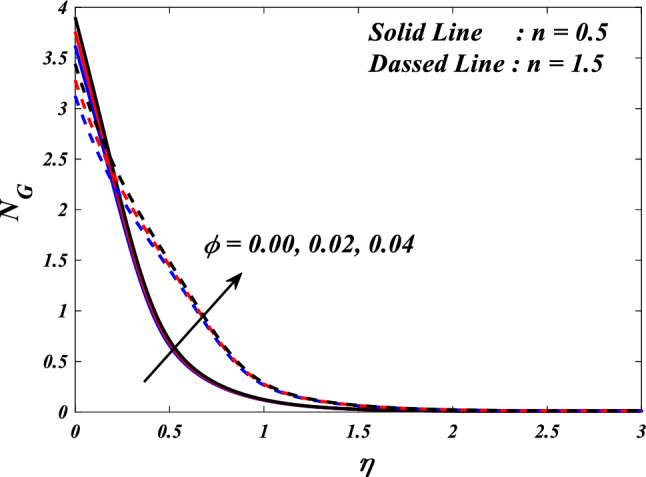
$${N}_{G}$$ vs $$\phi$$.

**Figure 27:**
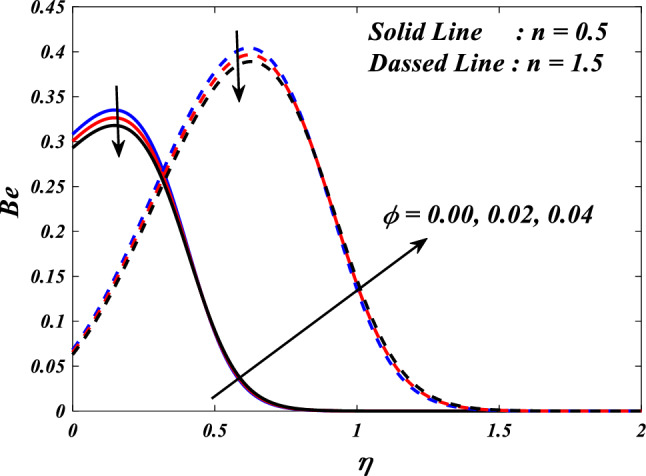
$$Be$$ vs $$\phi$$.

Figures 22 and 23 depict how the electric field parameter $\left(E=\mathrm{0.0,0.5,1.0}\right)$ affected the entropy generation $\left({N}_{G}\right)$ Bejan number $\left(Be\right)$ profiles of $n=0.5\&1.5$ respectively. It has been determined that ${N}_{G}$ intensifications for enlarged electric field parameter values in the case of $n=1.5$ and it is decreased for $n=0.5$. It has been determined that $Be$ decreases for higher electric field parameter $\left(E\right)$ values in both the cases $n=0.5\&1.5$ respectively. The foundational physical mechanism responsible for the reduction in entropy generation and Bejan number as a function of increasing electric field parameter is contingent upon the particular system under consideration and the characteristics of the electric field interaction. The temperature ratio parameter $\left({\theta }_{w}\right)$ effects on the ${N}_{G}$ and $Be$ profiles are shown in Figs. 24 and 25, respectively. It has been determined that ${N}_{G}$ and $Be$ intensifications for enlarged temperature ratio parameter $\left({\theta }_{w}\right)$ in the case of $n=0.5\&1.5$. The temperature ratio parameter displays how hot or cold certain system areas or components are in relation to one another. With an increase in the temperature disparity between these areas, a greater thermal gradient exists that propels the heat transfer process. The greater temperature disparity results in escalated entropy production caused by irreversible mechanisms. The Bejan number is a dimensionless parameter that quantifies the ratio of irreversibility to available energy in a given system. With an increase in the temperature ratio, the irreversibility connected to heat transfer and fluid flow become comparatively more significant in relation to the available energy. This results in an escalation of the Bejan number. Figures 26 and 27 depict how the nanoparticle volume fraction $\left(\phi \right)$ affected entropy generation $\left({N}_{G}\right)$, Bejan number $\left(Be\right)$ profiles of $n=0.5\&1.5$ respectively. It has been determined that $\left({N}_{G}\right)$ and $\left(Be\right)$ intensifications for enlarged nanoparticle volume fraction $\left(\phi \right)$. It is a common observation that the viscosity of the fluid-nanoparticle mixture tends to increase with an increase in the volume fraction of nanoparticles. The inclusion of nanoparticles has the potential to perturb the fluidic motion, leading to supplementary dissipation of energy via viscous mechanisms. The augmented viscous dissipation leads to an elevation in entropy generation within the system.

**Figure 28:**
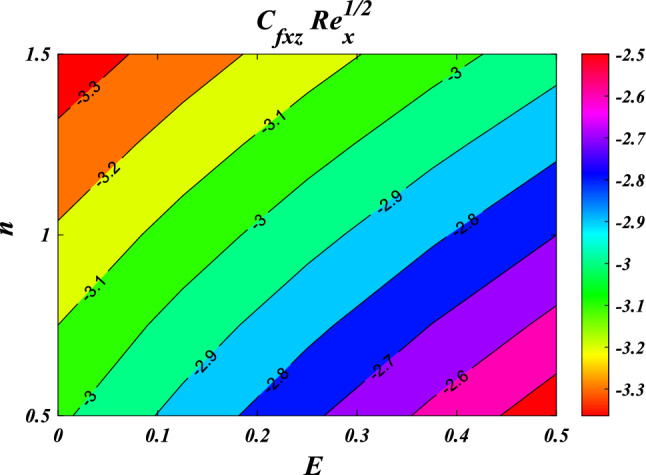
$${C}_{fxz}R{e}_{x}^{1/2}$$ vs $$E$$ and $$n$$.

**Figure 29:**
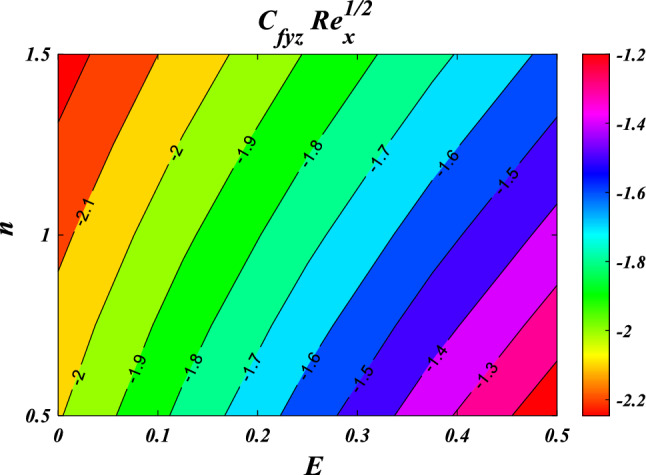
$${C}_{fyz}R{e}_{x}^{1/2}$$ vs $$E$$ and $$n$$.

**Figure 30:**
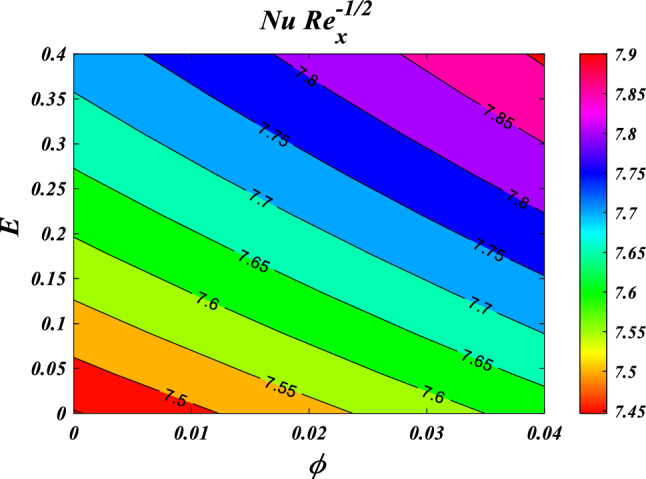
$$Nu{Re}_{x}^{-1/2}$$ vs $$\phi$$ and $$E$$.

**Figure 31:**
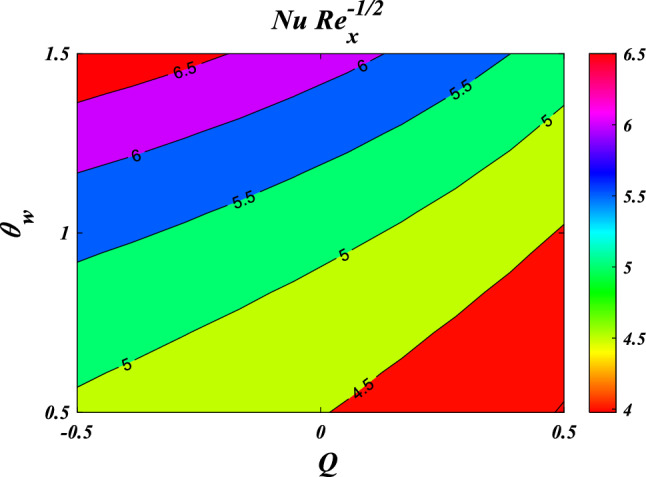
$$Nu{Re}_{x}^{-1/2}$$ vs $${\theta }_{w}$$ and $$Q$$.

**Figure 32:**
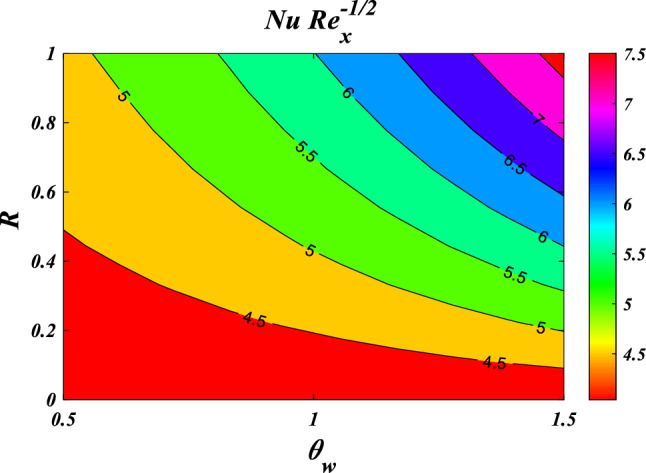
$$Nu{Re}_{x}^{-1/2}$$ vs $${\theta }_{w}$$ and $$R$$.

Figure 28 endorses the electric field parameter $\left(E\right)$ and velocity power index $\left(n\right)$ parameters on ${C}_{fxz}{Re}_{x}^{1/2}$. It is recognised that ${C}_{fxz}{Re}_{x}^{1/2}$ of tri-hybrid nano-fluid at the surface increases for amplifying $E$. It is shown that increasing $n$ values increases the ${C}_{fxz}{Re}_{x}^{1/2}$ of tri- hybrid nanofluid. Figure 29 encourages the effective use of the electric field parameter $\left(E\right)$ and the velocity power index $\left(n\right)$ on the coefficient of friction ${C}_{fyz}{Re}_{x}^{1/2}$. The amplification of $E$ results in an increase of the surface ${C}_{fyz}{Re}_{x}^{1/2}$ of tri-hybrid nano-fluid, as acknowledged. The data demonstrates that the augmentation of $n$ values results in an elevation of the ${C}_{fyz}{Re}_{x}^{1/2}$ of tri-hybrid nanofluid. Figure 30 is outlined to reveal the influence of electric field $\left(E\right)$ and heat source/sink $\left(Q\right)$ parameters on $Nu{Re}_{x}^{-1/2}$. It is discovered that decays on the Nusselt number of electric field $\left(E\right)$ and heat source/sink $\left(Q\right)$ parameters. The role of temperature ratio parameter $\left({\theta }_{w}\right)$ and heat source/sink parameter $\left(Q\right)$ on the $Nu{Re}_{x}^{-1/2}$ for the tri-hybrid nanofluid is explored through Fig. 31. It is manifest that improving the values of the $\left({\theta }_{w}\right)$ and $\left(Q\right)$ increments for the $Nu{Re}_{x}^{-1/2}$. Figure 32 is sketched to disclose the impact temperature ratio parameter $\left({\theta }_{w}\right)$ and thermal radiation parameter $\left(R\right)$ on $Nu{Re}_{x}^{-1/2}$. It is revealed that improving the values of the ${\theta }_{w}$ is increasing and $R$ initially increasing and then decrements for the $Nu{Re}_{x}^{-1/2}$.

## Conclusions

This analysis aims to determine the solar radiative effects on an EMHD micropolar hybrid nanofluid flow in a Non-Darcy 3-Dimensional slendering sheet with non-Fourier heat flux model and the influence of a uniform heat source. The main goal of this research is to depict the Silion Oil -based Silicon (Si) Magnesium oxide (MgO) and Titanium (Ti) nanoparticles flow properties after the mechanism. The impact of distinguished parameters like thermal relaxation, internal heat generation/ heat absorption, nanoparticle volume fraction, rate of heat transfer, skin friction factor and thermal radiation effects are analyzed. The non-linear thermal radiation properties of the nanofluid can be utilized in solar selective coatings. These coatings can be applied to solar panels or surfaces to selectively absorb or emit specific wavelengths of solar radiation, enhancing their overall energy conversion efficiency. The results are shown through two-dimensional graphs, contour figures and tables. The following significant findings emerged from this investigation:

- The artificial neural network model consumes less power for computing, is fast convergent, and doesn't need linearization.
- Higher values of the porosity parameter $\left(K\right)$ reduces the velocity $\left({f}{\prime}(\eta )\&{g}{\prime}(\eta )\right)$ profile but it is increases for the temperature $\left(\theta \left(\eta \right)\right)$ profile.
- The micro-rotation profiles $\left({{h}_{1}}{\prime}(\eta )\right)$ augmented by intensifying values vortex viscosity parameter $\left({R}_{1}\right)$.
- Higher values of electric field $\left(E\right)$ is decreased to the temperature $\left(\theta \left(\eta \right)\right)$ profile.
- It has been determined that entropy generation $\left({N}_{G}\right)$ and Bejan number $\left(Be\right)$ intensifications for enlarged nanoparticle volume fraction $\left(\phi \right)$.
- The skin frication ${C}_{fyz}{Re}_{x}^{1/2}$ is increases to progress the $n$ ands electric field $(E)$ parameters.
- Improving the values of the heat source $(Q)$ and temperature ratio ${(\theta }_{w})$ augmentations for the Nusselt number.

## List of symbols

$j$: Micro-inertia per unit mass (m^2^/kg)
$${F}_{s}$$: Inertia coefficient
$$K$$: Porosity parameter
*b*: Thermal accommodation coefficients
${k}_{1}$: Permeability of the porous medium
*T*: Temperature of the fluid $\left( {\text{K}} \right)$
$$R$$: Radiation parameter
$$n$$: Power index
$$Ec$$: Eckert number
$k^{*}$: Mean absorption coefficient
${h}_{2}^{*}$: Jump variable of temperature
$R_{3}$: Micro-inertia density parameter
$Re_{x}$: Local Reynolds number
${c}_{p}$: Fluid heat capacity $(J/kgK)$
$R_{1}$: Vortex viscosity parameter
${h}_{1}^{*}$: Dimensional velocity slip variable
${\lambda }_{T}$: Thermal relaxation time
$$l$$: Boundary parameter
$R_{2}$: Spin gradient viscosity parameter
$$u_{w} ,\,v_{w}$$: Stretching velocities (m/s)
$$Pr$$: Prandtl number
$$Be$$: Bejan number
$$M$$: Magnetic parameter
$E$: Electric field parameter
$$Br$$: Brinkman number
${N}_{G}$: Local entropy generation
k: Thermal conductivity $$(W/mK)$$
*u, v, w*: Components of velocities in *x*,* y*, and *z* directions respectively $\left( {\text{m/s}} \right)$
$T_{\infty }$: Temperature of the ambient fluid $\left( {\text{K}} \right)$
$T_{0}$: Reference temperature of the fluid $\left( {\text{K}} \right)$

## Greek letters

$\chi$: Spin gradient viscosity (N s/m^2^)
$\mu$: Dynamic viscosity (Pa s)
$$\kappa$$: Vortex viscosity $$\left( {{\text{m}}^{{2}} {\text{/s}}} \right)$$
${\alpha }_{1}$: Dimensionless ratio variable
${\tau }_{2}$: Temperature jump parameter
$\lambda$: Wall thickness variable
${\xi }_{1},{\xi }_{2}$: Constants (mean free path)
$\omega$: Micro rotation vector (rad/s)
$\omega_{1}$,$\omega_{2}$: Components of $\omega$ (rad/s)
$$\psi$$: Stream function
$\rho$: Density of the fluid $\left( {{\text{kg/m}}^{{3}} } \right)$
$$\sigma$$: Electrical conductivity $$\left( {\text{S/m}} \right)$$
$\sigma^{*}$: Stefan–Boltzmann constant
$$\zeta$$: Similarity variable
${\theta }_{w}$: Temperature ratio parameter
${\tau }_{1}$: Velocity slip variable
$\beta$: Stretching ratio parameter
$\Lambda$: Thermal relaxation time parameter
$\phi$: Nanoparticle volume fraction

## Subscripts

$$bf$$: Base fluid
MgO: Magnesium oxide
Ti: Titanium
Si: Silica
